# Concomitant use of direct oral anticoagulants and interacting antiarrhythmic drugs and the risk of stroke and bleeding among patients with non-valvular atrial fibrillation: a multinational cohort study

**DOI:** 10.1186/s12916-025-04464-6

**Published:** 2025-10-28

**Authors:** Fabian Maximilian Meinert, Jenny Dimakos, Ying Cui, Kristian B. Filion, Christel Renoux, Antonios Douros

**Affiliations:** 1 Institute of Clinical Pharmacology and Toxicology, Charité-Universitätsmedizin Berlin, Berlin, Germany; 2 Department of Medicine, McGill University, Montreal, QC Canada; 3 Centre for Clinical Epidemiology, Lady Davis Institute, Montreal, QC Canada; 4 Department of Epidemiology, Biostatistics and Occupational Health, McGill University, Montreal, QC Canada; 5 Department of Neurology and Neurosurgery, McGill University, Montreal, QC Canada

**Keywords:** Pharmacoepidemiology, Real-world evidence, Drug-drug interaction

## Abstract

**Background:**

Several antiarrhythmic drugs can interact with direct oral anticoagulants (DOACs) through pharmacokinetic mechanisms increasing DOAC levels. Our multinational cohort study assessed the effectiveness and safety of concomitant use of DOACs and interacting antiarrhythmic drugs among patients with non-valvular atrial fibrillation (NVAF).

**Methods:**

We used United Kingdom’s Clinical Practice Research Datalink and Quebec administrative claims data assembling two cohorts of patients with NVAF who initiated DOACs and added-on antiarrhythmic drugs. We assessed the risk of ischemic stroke and major bleeding associated with concomitant use of DOACs and interacting antiarrhythmic drugs (amiodarone, diltiazem, dronedarone, verapamil) versus concomitant use of DOACs and non-interacting antiarrhythmic drugs (flecainide, propafenone, sotalol) using an as-treated exposure definition. Cox models yielded hazard ratios (HRs) and 95% confidence intervals (CIs) after inverse-probability-of-treatment-weighting. We pooled site-specific estimates together using random-effects models. Secondary analyses stratified by age, sex, and individual DOACs.

**Results:**

Our study cohort included 54,078 NVAF patients initiating DOACs and adding-on antiarrhythmic drugs. Concomitant use of DOACs and interacting antiarrhythmic drugs versus concomitant use of DOACs and non-interacting antiarrhythmic drugs was not associated with the risk of ischemic stroke (pooled HR, 1.04; 95% CI, 0.88–1.21; I^2^ = 0%) but with an increased risk of major bleeding (pooled HR, 1.30; 95% CI, 1.19–1.41; I^2^ = 58%), especially among patients < 70 years (pooled HR, 1.56; 95% CI, 1.31–1.86; I^2^ = 0%). There was no effect modification by sex or individual DOAC.

**Conclusions:**

Concomitant use of interacting antiarrhythmic drugs does not seem to affect the effectiveness of DOACs but may increase their risk of major bleeding.

**Supplementary Information:**

The online version contains supplementary material available at 10.1186/s12916-025-04464-6.

## Background

Several antiarrhythmic drugs interact with direct oral anticoagulants (DOACs) via pharmacokinetic mechanisms such as the inhibition of the cytochrome P450 3A4 (CYP3A4) enzyme or the permeability glycoprotein (p-pg) transporter (https://drug-interactions.medicine.iu.edu/MainTable.aspx, [1–3]). These pharmacokinetic interactions lead to increases in DOAC systemic levels of varying magnitude (up to 143% increased dabigatran concentration upon co-administration of verapamil [4]), which could then affect their effectiveness and safety [5]. Given the common concomitant use of DOACs and antiarrhythmic drugs especially among patients with non-valvular atrial fibrillation (NVAF) [1], an assessment of the clinical effects of these interactions is important.

Post-hoc analyses of randomized trials compared the risk of ischemic stroke and major bleeding between DOACs and the therapeutic alternatives vitamin K antagonists (VKAs) among patients with NVAF receiving certain antiarrhythmic drugs [6, 7]. However, the design of these studies did not allow the estimation of the impact of the aforementioned interactions on the effectiveness and safety of DOACs directly, but only in relation to VKAs. Therefore, observational studies have aimed to fill in this knowledge gap [8–12]. The results on major bleeding have been conflicting, whereas only two studies assessed the risk of ischemic stroke [8, 9]. Moreover, methodological limitations such as strong confounding due to use of inadequate comparators [8, 10–12], and selection bias [10–12] render the interpretation of most findings challenging.

Given the inconsistencies and limitations of existing literature, we conducted a large, multi-national cohort study to assess whether the concomitant use of DOACs and pharmacokinetically interacting antiarrhythmic drugs is associated with the risk of ischemic stroke or major bleeding, when compared to the concomitant use of DOACs and non- or weakly interacting antiarrhythmic drugs.

## Methods

### Data source

We conducted our population-based cohort study using the United Kingdom’s (UK’s) Clinical Practice Research Datalink (CPRD) Aurum and the Canadian Régie de l’assurance maladie du Québec (RAMQ). The CPRD is the world’s largest primary care database containing records of 60 million patients seen across > 2,000 general practices, and is representative of the general UK population (https://cprd.com/, [13]). All prescriptions issued by general practitioners are recorded; the CPRD also contains clinical measures (e.g. blood pressure [BP]), laboratory findings, anthropometric measures (e.g. body mass index [BMI]), and lifestyle variables (e.g. smoking) (https://cprd.com/). Moreover, the CPRD records diagnoses based on SNOMED Clinical terms (structured clinical vocabulary for electronic health records) and local EMIS® Web codes (coding system including clinical events, online test requests, test results, and prescriptions), systems with greater granularity than the International Classification of Diseases (ICD) ([13, https://www.emishealth.com/about-us). The CPRD was linked to the Hospital Episode Statistics database, which contains hospital admissions, inpatient procedures, and discharge diagnoses (coded using ICD-10), the Office for National Statistics database, which contains vital statistics data including date, place and underlying cause of death (coded via ICD-10), and the Index of Multiple Deprivation (IMD), a proxy of socioeconomic status.

The RAMQ includes data on all residents of Québec who are aged ≥ 65 years or have no private insurance plans or are recipients of financial assistance. The RAMQ includes demographic characteristics, outpatient diagnoses (coded using ICD-9/-10), outpatient procedures, and dispensed drug prescriptions. The RAMQ was linked to the Maintenance et exploitation des données pour l’étude de la clientèle hospitalière database, which contains hospital admissions, inpatient procedures, and discharge diagnoses (coded using ICD-10) and to the Institut de la statistique du Québec (ISQ) database, which contains vital statistics data (https://www.ramq.gouv.qc.ca/en). The study protocol was approved by the independent Scientific Advisory Committee of the CPRD (protocol number: 23_002915), the ISQ, and the Research Ethics Board of the Jewish General Hospital Montreal, Canada.

### Study cohort

We included all patients newly diagnosed with NVAF between January 2011 (when dabigatran was the first DOAC approved for stroke prevention in NVAF in the UK and Canada) and June 2020 (latest date of CPRD data availability) or December 2020 (latest date of RAMQ data availability). We excluded all patients aged < 18 years, with database history < 365 days, diagnosed with valvular heart disease at any time before or with hyperthyroidism in the year prior, or prescribed a DOAC in the year prior. Then, we identified all patients who initiated a DOAC (apixaban, dabigatran, edoxaban, rivaroxaban) after the NVAF diagnosis, excluding patients with a prescription for an antiarrhythmic drug in the year prior.

Our study cohort comprised initiators of DOACs who added on an antiarrhythmic drug (sodium channel blockers: flecainide, propafenone; potassium channel blockers: amiodarone, dronedarone, sotalol; calcium channel blockers: verapamil, diltiazem) either on the same date as the initiation of a DOAC or at some point later on but overlapping with DOAC use. Cohort entry was the date of the initiation of concomitant use (study design in Additional file 1: Figure S1). Patients were followed until an event (defined below), treatment switch or discontinuation (defined below), administrative censoring, death, or end of the study period, whichever occurred first. Each patient was allowed to contribute only one episode of concomitant use to the study.

### Exposure definition

By design, we had two exposure groups: (i) patients with concomitant use of DOACs and antiarrhythmic drugs with moderate-to-strong CYP3A4 or p-gp inhibitory potential (amiodarone, diltiazem, dronedarone, verapamil (https://drug-interactions.medicine.iu.edu/MainTable.aspx, [2, 3]); from now on ‘interacting antiarrhythmic drugs’ for brevity), and (ii) patients with concomitant use of DOACs and antiarrhythmic drugs with no or weak CYP3A4 or p-gp inhibitory potential (flecainide, propafenone, sotalol [14]; from now on ‘non-interacting antiarrhythmic drugs’ for brevity). We used an as-treated exposure definition, where patients were considered continuously co-exposed if the duration of successive prescriptions of DOACs and of antiarrhythmic drugs were overlapping each other. We allowed for a 30-day grace period in the event of non-overlapping successive prescriptions to account for less-than-perfect adherence. Treatment switch was defined as a prescription for a non-interacting antiarrhythmic drug among patients concomitantly using DOACs and interacting antiarrhythmic drugs or vice versa. Switches between different interacting antiarrhythmic drugs (e.g., from diltiazem to verapamil) or between different non-interacting antiarrhythmic drugs (e.g., from flecainide to sotalol) were allowed.

### Outcome definition

Primary effectiveness outcome was ischemic stroke, defined as a composite endpoint of hospitalization with ischemic stoke, transient ischemic attack (TIA), or systemic embolism (SE). Primary safety outcome was major bleeding, defined as hospitalization with bleeding. Secondary safety outcomes included different subtypes of major bleeding (intracranial hemorrhage [ICH], gastrointestinal bleeding [GIB], other major bleeding) (ICD codes in Additional file 1: Table S1.

### Covariates

We adjusted for the following potential confounders measured at cohort entry: time since NVAF diagnosis, age (modelled flexibly using restricted cubic splines to account for potential non-linear associations with the study outcomes), and sex. We also adjusted for the following comorbidities, diagnosed at any time before cohort entry: alcohol-related disorders, hypertension, prior ischemic stroke/TIA/SE, congestive heart failure, coronary artery disease, peripheral vascular disease, major bleeding, type 2 diabetes mellitus, liver disease, and renal disease. Moreover, we included cancer (other than non-melanoma skin cancer) diagnosed in the year before cohort entry. Furthermore, we adjusted for use of VKAs, antiplatelet agents, and selective serotonin reuptake inhibitors in the year before cohort entry. As a proxy for overall health, we used the number of hospitalizations in the year before cohort entry. In the CPRD, we also considered smoking status (current, former, never, unknown), BMI category (< 25 kg/m^2^, 25–29 kg/m^2^, ≥ 30 kg/m^2^, unknown), BP levels (systolic BP ≥ 130 mmHg or diastolic BP ≥ 80 mmHg, systolic BP < 130 mmHg and diastolic BP < 80 mmHg, unknown), and socioeconomic status (IMD quintiles), using the last measurement before cohort entry. In the RAMQ, we also considered in the number of non-anticoagulant drugs in the year before cohort entry. For the analyses on major bleeding, we additionally adjusted for use of non-steroidal anti-inflammatory drugs (NSAIDs), proton pump inhibitors, and H_2_ blockers in the year before cohort entry.

### Statistical analyses

Crude incidence rates with 95% confidence intervals (CIs) for the study outcomes for each exposure group were calculated assuming a Poisson distribution. We applied propensity score (PS) based inverse-probability-of-treatment-weighting (IPTW) for confounding control. Multivariable logistic regression calculated PS that predicted the probability of receiving the exposure of interest (concomitant use of DOACs and interacting antiarrhythmic drugs) versus the reference category, (concomitant use of DOACs and non-interacting antiarrhythmic drugs), conditional on all previously listed covariates. We calculated PS separately for new users of DOACs and for prevalent users of DOACs given the potential heterogeneity related to the type of DOAC user and then pooled the strata-specific PS together. Imbalances in covariates after IPTW were assessed using standardized differences; those with values ≥ 0.1 were deemed clinically meaningful and were included in the outcome models. Hazard ratios (HRs) and 95% confidence intervals (CIs) of the study outcomes were estimated using Cox proportional hazards models. Site-specific estimates were pooled together using random-effects models [15].

### Secondary analyses

To assess potential effect measure modification we conducted several secondary analyses, thereby repeating PS based IPTW within each stratum. First, we stratified by age (≥ 70 versus < 70 years) and sex. Second, we stratified by baseline outcome risk using the CHA_2_DS_2_-VASc score (congestive heart failure, hypertension, age ≥ 75 years, diabetes mellitus, stroke, vascular disease, age 65–74 years, sex) for ischemic stroke and a modified version of the HAS-BLED score (hypertension, abnormal renal or liver function, stroke, bleeding, elderly, drugs or excess alcohol use) for major bleeding. Third, we stratified by the individual DOACs rivaroxaban and apixaban, the two most commonly used compounds for stroke prevention in NVAF [16, 17]. Finally, we stratified by type of DOAC use (new versus prevalent use).

### Sensitivity analyses

We performed seven pre-planned sensitivity analyses. First, to assess potential exposure misclassification, we used a 15-day grace period between non-overlapping successive prescriptions. Second, to assess potential outcome misclassification, we used a stricter outcome definition based on hospitalization codes in primary position only. Third, we additionally included fatal events in the definition of the study outcomes. Fourth, to eliminate residual confounding due to prior events, we excluded patients with a history of ischemic stroke or major bleeding. Fifth, we used multiple imputation for missing values for BMI and BP (only applicable in the CPRD). Sixth, to address potential selection bias due to informative censoring, we used an intention-to-treat exposure definition imposing a maximum follow-up of 1 year. Finally, as an alternate means to control for informative censoring, we used inverse-probability-of-censoring-weighting including all previously listed covariates as time-fixed covariates and alcohol-related disorders, use of antiplatelet agents, and use of NSAIDs (the latter only for major bleeding) as time-varying covariates. Extreme weights were truncated using the 99th percentile as cut-off. All analyses were conducted with SAS 9.4 software (SAS Institute, Cary, NC).

## Results

Overall, our study cohort consisted of 54,078 patients with NVAF (16,820 in the CPRD and 37,258 in the RAMQ) who initiated DOACs (apixaban 48%, dabigatran 12%, edoxaban 2%, rivaroxaban 38%) and added-on antiarrhythmic drugs (Additional file 1: Figure S2). Of those, 46% (*n* = 24,843) initiated antiarrhythmic drugs on the same date as DOACs and 54% (*n* = 29,235) initiated antiarrhythmic drugs at a later point but with overlapping DOAC use. Baseline characteristics of patients are shown in Additional file 1: Tables S2-S3. Patients in the RAMQ were older and more likely to have been diagnosed with cardiometabolic comorbidities than in the CPRD, while patients in the CPRD were more likely to have been diagnosed with renal disease. Patients with concomitant use of DOACs and interacting antiarrhythmic drugs were more likely to have been diagnosed with cardiometabolic comorbidities and renal disease than those with concomitant use of DOACs and non-interacting antiarrhythmic drugs. After IPTW, all baseline characteristics were well-balanced.

Compared to concomitant use of DOACs and non-interacting antiarrhythmic drugs, concomitant use of DOACs and interacting antiarrhythmic drugs was not associated with the risk of ischemic stroke (pooled weighted risk difference per 1000 person-years, 0.41; 95% CI, −1.41–2.23; I^2^ = 0%/pooled HR, 1.04; 95% CI, 0.88–1.21; I^2^ = 0%). However, there was an association with an increased risk of major bleeding (pooled weighted risk difference per 1000 person-years, 10.86; 95% CI, 7.39–14.33; I^2^ = 74%/pooled HR, 1.30; 95% CI, 1.19–1.41; I^2^ = 58%) (Table 1) (cumulative incidences in Additional file 1: Figure S3, reasons for censoring in Additional file 1: Table S4). This effect was led by increased risks of GIB (pooled HR, 1.45; 95% CI, 1.26–1.66; I^2^ = 59%) and other major bleeding (pooled HR, 1.21; 95% CI, 1.09–1.36; I^2^ = 24%), but not ICH (pooled HR, 0.81; 95% CI, 0.59–1.12; I^2^ = 0%) (Table 2).

**Table 1:** Risk of ischemic stroke and major bleeding associated with use of DOACs and interacting antiarrhythmics versus use of DOACs and non-interacting antiarrhythmics among patients with NVAF

	**N** **Patients**	**N** **Events**	**N** **PY**	**IR**^**a**^	**Crude HR** **(95% CI)**	**IPTW HR (95% CI)**	**Pooled HR** **(95% CI)**	**I**^**2**^
Ischemic stroke/TIA/SE							1.04 (0.88–1.21)	0%
CPRD								
DOACs + interacting antiarrhythmics	12,487	92	10,119	9.09	1.97 (1.16–3.35)	1.04 (0.69–1.58)		
DOACs + non-interacting antiarrhythmics	4,333	16	3,484	4.59	1.00 (reference)	1.00 (reference)		
RAMQ								
DOACs + interacting antiarrhythmics	28,504	616	43,983	14.01	1.59 (1.31–1.93)	1.04 (0.87–1.23)		
DOACs + non-interacting antiarrhythmics	8,754	123	14,172	8.68	1.00 (reference)	1.00 (reference)		
Major bleeding							1.30 (1.19–1.41)	58%
CPRD								
DOACs + interacting antiarrhythmics	12,487	408	9,931	41.08	1.53 (1.22–1.92)	1.12 (0.92–1.38)		
DOACs + non-interacting antiarrhythmics	4,333	92	3,431	26.82	1.00 (reference)	1.00 (reference)		
RAMQ								
DOACs + interacting antiarrhythmics	28,504	2,373	42,477	55.87	2.03 (1.82–2.27)	1.34 (1.22–1.48)		
DOACs + non-interacting antiarrhythmics	8,754	375	13,949	26.88	1.00 (reference)	1.00 (reference)		

*Abbreviations*: *PY* patient years, *IR* incidence rate, *HR* hazard ratio, *CI* confidence interval, *IPTW* inverse probability of treatment weighting, *DOACs* direct oral anticoagulants, *NVAF* non-valvular atrial fibrillation, *TIA* transient ischemic attack, *SE* systemic embolism, *CPRD* Clinical Practice Research Datalink, *RAMQ* Régie de l’Assurance-Maladie du Québec

^a^IR per 1,000 PY

**Table 2:** Risk of different subtypes of major bleeding associated with the use of DOACs and interacting antiarrhythmics versus use of DOACs and non-interacting antiarrhythmics among patients with NVAF

	**N** **Patients**	**N** **Events**	**N** **PY**	**IR**^**a**^	**Crude HR** **(95% CI)**	**IPTW HR (95% CI)**	**Pooled HR** **(95% CI)**	**I**^**2**^
Intracranial hemorrhage							0.81 (0.59–1.12)	0%
CPRD								
DOACs + interacting antiarrhythmics	12,487	14	10,161	1.38	1.21 (0.40–3.67)	0.64 (0.27–1.54)		
DOACs + non-interacting antiarrhythmics	4,333	S^b^	S^b^	1.15	1.00 (reference)	1.00 (reference)		
RAMQ								
DOACs + interacting antiarrhythmics	28,504	126	44,394	2.84	1,54 (1.01–2.35)	0.85 (0.60–1.20)		
DOACs + non-interacting antiarrhythmics	8,754	26	14,295	1,82	1.00 (reference)	1.00 (reference)		
Gastrointestinal bleeding							1.45 (1.26–1.66)	59%
CPRD								
DOACs + interacting antiarrhythmics	12,487	160	10,079	15.87	1.49 (1.04–2.13)	1.15 (0.83–1.59)		
DOACs + non-interacting antiarrhythmics	4,333	S^b^	S^b^	10.69	1.00 (reference)	1.00 (reference)		
RAMQ								
DOACs + interacting antiarrhythmics	28,504	1,090	43,565	25.02	2.25 (1.90–2.67)	1.52 (1.31–1.77)		
DOACs + non-interacting antiarrhythmics	8,574	154	14,157	10.88	1.00 (reference)	1.00 (reference)		
Other major bleeding							1.21 (1.09–1.36)	24%
CPRD								
DOACs + interacting antiarrhythmics	12,487	249	10,009	24.88	1.59 (1.18–2.13)	1.06 (0.83–1.37)		
DOACs + non-interacting antiarrhythmics	4,333	54	3,462	15.60	1.00 (reference)	1.00 (reference)		
RAMQ								
DOACs + interacting antiarrhythmics	28,504	1,314	43,127	30.41	1.90 (1.64–2.19)	1.25 (1.11–1.42)		
DOACs + non-interacting antiarrhythmics	8,754	221	14,076	15.70	1.00 (reference)	1.00 (reference)		

*Abbreviations*: *PY* patient years, *IR* incidence rate, *HR* hazard ratio, *CI* confidence interval, *IPTW* inverse probability of treatment weighting, *DOACs* direct oral anticoagulants, *NVAF* non-valvular atrial fibrillation, *TIA* transient ischemic attack, *SE* systemic embolism, *CPRD* Clinical Practice Research Datalink, *RAMQ* Régie de l’Assurance-Maladie du Québec

^a^IR per 1,000 PY

^b^Suppressed due to small numbers (< 5) as per confidentiality agreement with the CPRD

**Fig. 1:**
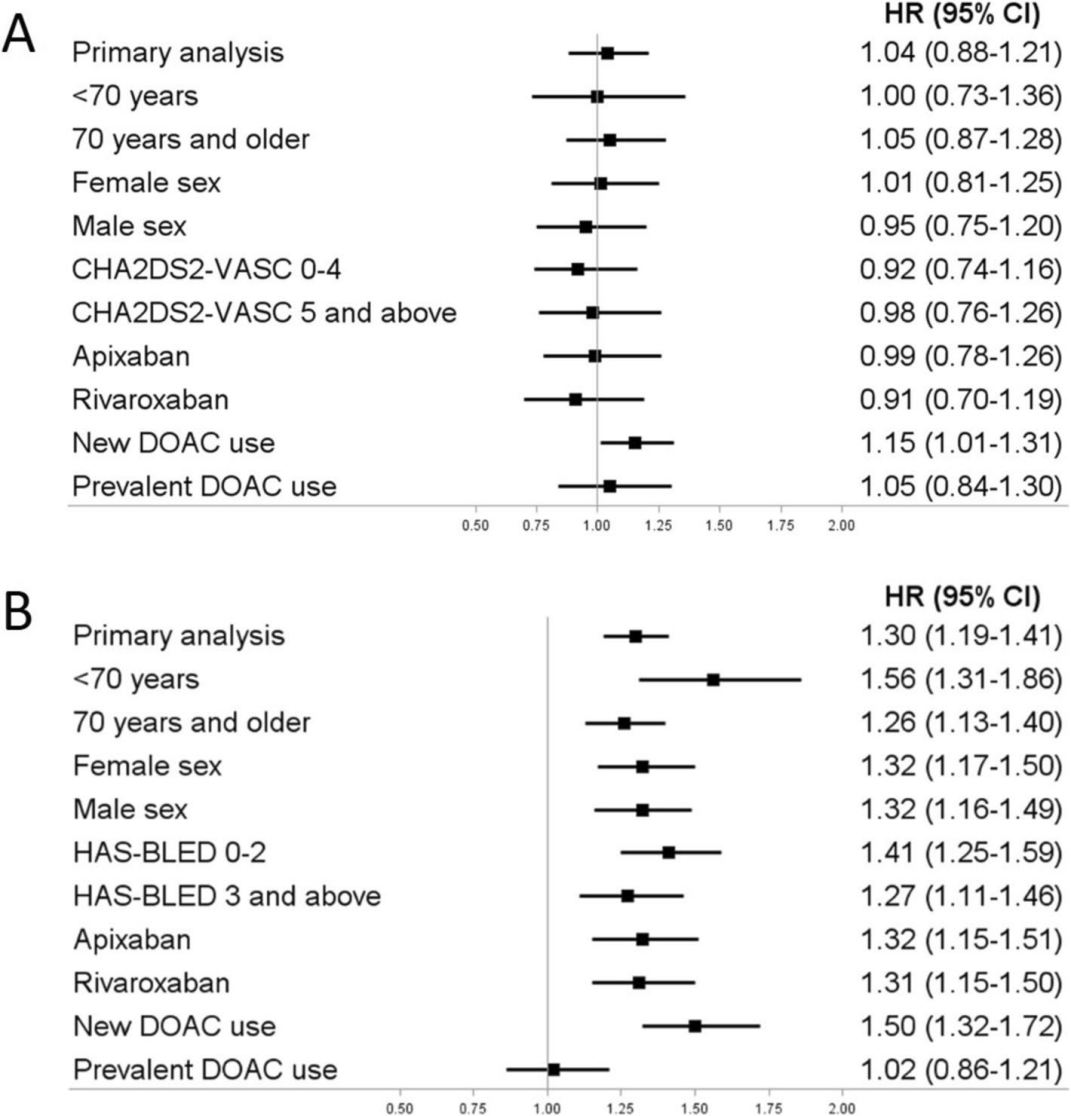
Forest plots summarizing the secondary analyses for both study outcomes. Panel **A** Ischemic stroke. Panel **B** Major bleeding. Abbreviations: CHA_2_DS_2_-VASc, congestive heart failure, hypertension, age ≥ 75 years, diabetes mellitus, stroke, vascular disease, age 65–74 years, sex; HAS-BLED, hypertension, abnormal renal or liver function, stroke, bleeding, elderly, drugs or excess alcohol use; DOACs, direct oral anticoagulants; HR, hazard ratio; CI, confidence interval

**Fig. 2:**
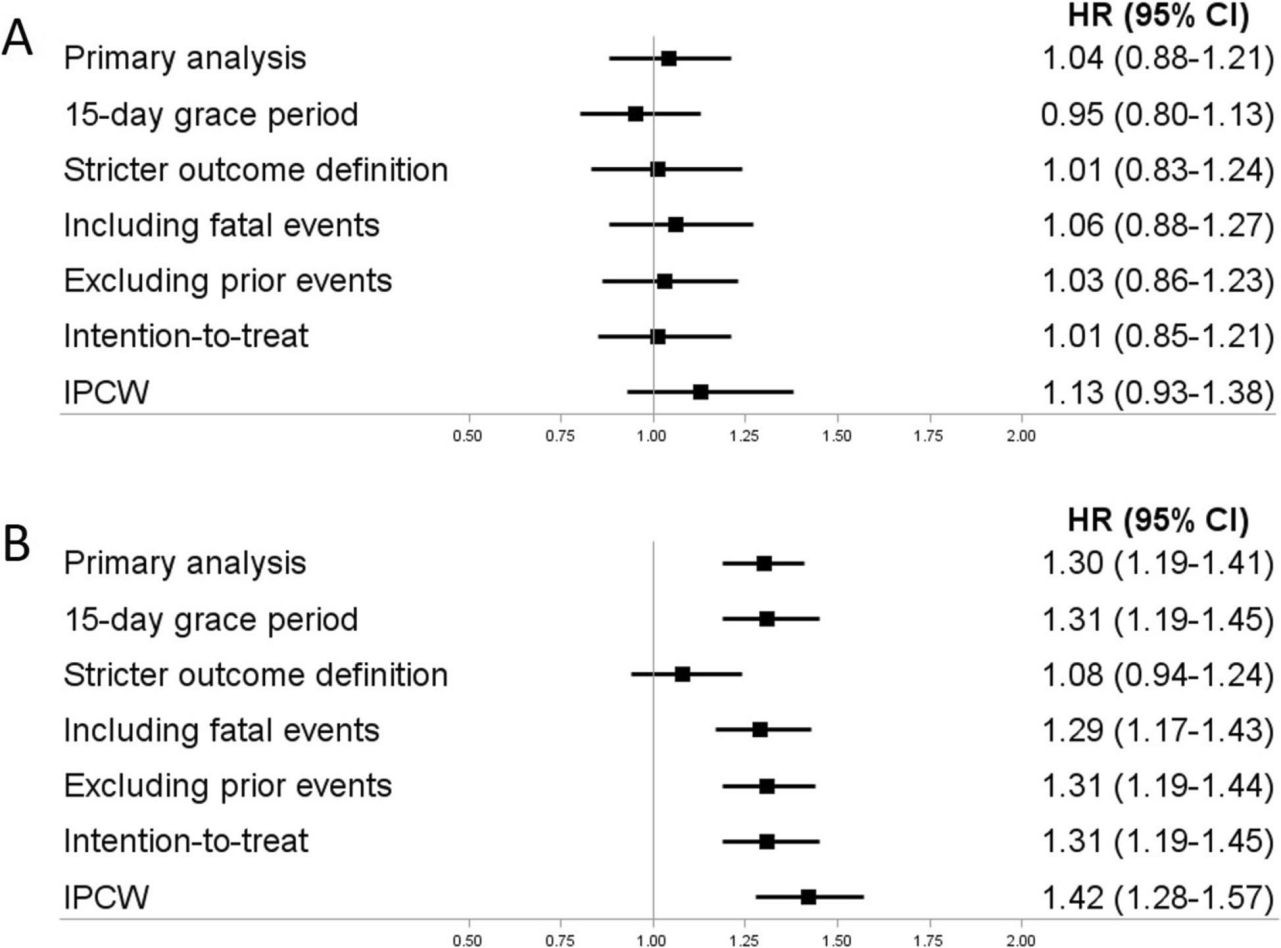
Forest plots summarizing the sensitivity analyses for both study outcomes. Panel **A** Ischemic stroke. Panel **B** Major bleeding. Abbreviations: IPCW, inverse probability of censoring weighting, HR, hazard ratio; CI, confidence interval

In secondary analyses (summarized in Fig. 1), we did not observe any major effect modifications by age, sex, baseline thrombotic risk, individual DOACs, or type of DOAC use for ischemic stroke (Additional file 1: Tables S5-S6) or by sex, baseline bleeding risk, or individual DOACs for major bleeding (Additional file 1: Tables S7-S8). However, there was an effect modification by age, with the increased risk of major bleeding associated with concomitant use of DOACs and interacting antiarrhythmic drugs being more pronounced among patients aged < 70 years (pooled HR, 1.56; 95% CI, 1.31–1.86; I^2^ = 0%) than those aged ≥ 70 years (pooled HR, 1.26; 95% CI, 1.13–1.40; I^2^ = 44%). Moreover, the increased risk of major bleeding was observed among patients initiating interacting antiarrhythmic drugs on the same day as DOACs (pooled HR, 1.50; 95% CI, 1.32–1.72; I^2^ = 0%) but not among those initiating interacting antiarrhythmic drugs later on (pooled HR, 1.02; 95% CI, 0.86–1.21; I^2^ = 0%). In sensitivity analyses (summarized in Fig. 2 and shown in detail in Additional file 1: Tables S9-S10), the results were consistent with those from the primary analyses both for ischemic stroke (HRs from 0.95 to 1.13) and major bleeding (HRs from 1.08 to 1.42), albeit with some variation in the effect estimates.

## Discussion

Our large, multinational cohort study of over 50,000 patients with NVAF showed that concomitant use of DOACs and interacting antiarrhythmic drugs was not associated with the risk of ischemic stroke, when compared to concomitant use of DOACs and non-interacting antiarrhythmic drugs. However, there was an association with an increased risk of major bleeding, which was driven by GIB and non-GIB, non-ICH major bleeding. Moreover, the increased risk of major bleeding was stronger among patients aged < 70 years and among those co-initiating antiarrhythmic drugs and DOACs.

Our observation that concomitant use of DOACs and interacting antiarrhythmic drugs is associated with an increased risk of major bleeding is in line with the known pharmacokinetic features of these drug classes. Indeed, several antiarrhythmic drugs inhibit CYP3A4 and p-gp, and this inhibition can lead to elevated systemic levels of DOACs [5, 18]. Therefore, our findings support the notion that the increase in the concentration of DOACs translates into a higher risk of DOAC related bleeding.

There was some effect heterogeneity regarding the subtypes of major bleeding, with concomitant use of DOACs and interacting antiarrhythmic drugs being associated with an increased risk of GIB and non-GIB, non-ICH major bleeding but not ICH. A potential explanation may be related to the favorable safety profile of DOACs with respect to ICH [19], which seems to be retained even in case of elevated systemic drug levels. We also observed that the increased risk of major bleeding was stronger among patients aged < 70 years than those aged ≥ 70 years. This may be related to the general decrease in metabolic functions including the CYP3A4 and p-gp activity over the years [20], which could limit the clinical impact of CYP3A4 or p-gp mediated interactions among older adults.

The increase in the risk of major bleeding was driven by patients who initiated DOACs and antiarrhythmic drugs on the same day. Overall, co-initiators had higher incidence rates of major bleeding than patients who initiated antiarrhythmic drugs at a later point, possibly reflecting the higher clinical risk in cases of NVAF where early rhythm and rate control is required. Hence, our observations could be consistent with an effect modification of the pharmacologic interaction between DOACs and antiarrhythmic drugs by underlying NVAF severity. That being said, since NVAF severity was not directly measured, this hypothesis requires confirmation in future studies.

Prior observational studies assessing the clinical effects of concomitant use of DOACs and interacting antiarrhythmic drugs yielded conflicting findings regarding the risk of major bleeding (HRs between 0.77 and 2.87) [8–12] and inconclusive findings regarding the risk of ischemic stroke (numerically decreased effect estimates not reaching statistical significance: HRs, 0.80 and 0.87) [8, 9]. A major limitation of most studies was the implementation of inadequate comparators such as use of DOACs alone [11] or concomitant use of DOACs and medications not primarily used as antiarrhythmics such as amlodipine [10] or metoprolol [8, 10]. Importantly, inadequate comparators can exacerbate confounding, a bias that presents an inherent challenge in pharmacoepidemiology in general [21] and in the setting of drug-drug interactions in particular [22].

Our study has several strengths. First, the large sample size allowed the estimation of relatively precise effect estimates not only for major bleeding but also for ischemic stroke, thus overcoming a limitation of previous studies [10, 23]. Moreover, it enabled the implementation of several secondary analyses that assessed potential effect modifications in clinically important subgroups. Second, using two electronic healthcare databases from two different countries and healthcare systems likely maximized the generalizability of our study findings. Finally, the application of a common, prespecified protocol across databases maximized methodological homogeneity and enabled the pooling of site-specific estimates.

The study has also some limitations. First, due to the nature of electronic healthcare data, we were not able to measure the actual intake of medications. Instead, we used issued drug prescriptions (CPRD) or drug dispensations (RAMQ) as proxies of drug use. To account for potential exposure misclassification, we performed a sensitivity analysis with an alternate grace period that corroborated the findings from the primary analysis. Second, outcome misclassification is possible. However, sensitivity analyses using stricter or expanded outcome definitions led to consistent findings. Third, despite our best efforts to minimize confounding using an active comparator and PS based IPTW, residual confounding due to unmeasured confounders such as NVAF severity or poorly captured confounders such as drug dose cannot be excluded.

## Conclusions

Our multinational cohort study provides important evidence on the impact of the pharmacokinetic interactions between certain antiarrhythmic drugs and DOACs in the overall NVAF population and in clinically important subgroups. Specifically, while these interactions do not seem to affect the effectiveness of DOACs, they do affect their safety with increases in the relative and absolute risk of major bleeding of clinically relevant magnitude. Therefore, our findings support caution when concomitant use of DOACs and pharmacologically interacting antiarrhythmic drugs is required.

## Supplementary Information

Additional file 1: Figure S1. Study design. Figure S2. Flowchart illustrating the construction of the study cohort. Figure S3. Cumulative incidences of the study outcomes. Table S1. ICD-10 codes for the definition of ischemic stroke and major bleeding. Table S2. Baseline characteristics of patients in the CPRD. Table S3. Baseline characteristics of patients in the RAMQ. Table S4. Reasons for censoring. Table S5. Risk of ischemic stroke associated with concomitant use of DOACs and interacting antiarrhythmics compared with concomitant use of DOACs and non-interacting antiarrhythmics among patients with NVAF (stratification by demographics). Table S6. Risk of ischemic stroke associated with concomitant use of DOACs and interacting antiarrhythmics compared with concomitant use of DOACs and non-interacting antiarrhythmics (stratification by baseline risk, individual DOACs, and type of DOAC use). Table S7. Risk of major bleeding associated with concomitant use of DOACs and interacting antiarrhythmics compared with concomitant use of DOACs and non-interacting antiarrhythmics (stratification by demographics). Table S8. Risk of major bleeding associated with concomitant use of DOACs and interacting antiarrhythmics compared with concomitant use of DOACs and non-interacting antiarrhythmics (stratification by baseline risk, individual DOACs, and type of DOAC use). Table S9. Risk of ischemic stroke associated with concomitant use of DOACs and interacting antiarrhythmics compared with concomitant use of DOACs and non-interacting antiarrhythmics (sensitivity analyses). Table S10. Risk of major bleeding associated with concomitant use of DOACs and interacting antiarrhythmics compared with concomitant use of DOACs and non-interacting antiarrhythmics (sensitivity analyses).

## Data Availability

The data for this study are available from the data custodians of the UK CPRD and the RAMQ. Restrictions apply to the availability of these data, which were used under license for this study.

## Abbreviations

DOACs: Direct oral anticoagulants
CYP3A4: Cytochrome P450 3A4
p-gp: Permeability glycoprotein
NVAF: Non-valvular atrial fibrillation
VKAs: Vitamin K antagonists
UK: United Kingdom
CPRD: Clinical Practice Research Datalink
RAMQ: Régie de l’assurance maladie du Québec
BP: Blood pressure
BMI: Body mass index
ICD: International Classification of Diseases
IMD: Index of Multiple Deprivation
MED-ÉCHO: Maintenance et exploitation des données pour l’étude de la clientèle hospitalière
ISQ: Institut de la statistique du Québec
TIA: Transient ischemic attack
SE: Systemic embolism
ICH: Intracranial hemorrhage
GIB: Gastrointestinal bleeding
NSAIDs: Non-steroidal anti-inflammatory drugs
CIs: Confidence intervals
PS: Propensity score
IPTW: Inverse-probability-of-treatment-weighting
HRs: Hazard ratios
CHA_2_DS_2_-VASc: Congestive heart failure, hypertension, age ≥ 75 years, diabetes mellitus, stroke, vascular disease, age 65–74 years, sex
HAS-BLED: Hypertension, abnormal renal or liver function, stroke, bleeding, elderly, drugs or excess alcohol use

## References

[1] Writing Committee M, Joglar JA, Chung MK, et al. 2023 ACC/AHA/ACCP/HRS guideline for the diagnosis and management of atrial fibrillation: a report of the American College of Cardiology/American Heart Association Joint Committee on clinical practice guidelines. J Am Coll Cardiol. 2024;83(1):109–279. 38043043 10.1016/j.jacc.2023.08.017 PMC11104284

[2] Ohyama K, Nakajima M, Suzuki M, Shimada N, Yamazaki H, Yokoi T. Inhibitory effects of amiodarone and its N-deethylated metabolite on human cytochrome P450 activities: prediction of in vivo drug interactions. Br J Clin Pharmacol. 2000;49(3):244–53. 10718780 10.1046/j.1365-2125.2000.00134.x PMC2014912

[3] Hong Y, Chia YM, Yeo RH, et al. Inactivation of human cytochrome P450 3A4 and 3A5 by dronedarone and N-desbutyl dronedarone. Mol Pharmacol. 2016;89(1):1–13. 26490246 10.1124/mol.115.100891

[4] Härtter S, Sennewald R, Nehmiz G, Reilly P. Oral bioavailability of dabigatran etexilate (Pradaxa®) after co-medication with verapamil in healthy subjects. Br J Clin Pharmacol. 2013;75(4):1053–62. 22946890 10.1111/j.1365-2125.2012.04453.x PMC3612723

[5] Lin SY, Liu YB, Ho LT, et al. Impact of amiodarone on plasma concentration of direct oral anticoagulant in patients with atrial fibrillation. J Formos Med Assoc. 2023;122(8):776–84. 36890017 10.1016/j.jfma.2023.02.012

[6] Flaker G, Lopes RD, Hylek E, et al. Amiodarone, anticoagulation, and clinical events in patients with atrial fibrillation: insights from the ARISTOTLE trial. J Am Coll Cardiol. 2014;64(15):1541–50. 25301455 10.1016/j.jacc.2014.07.967

[7] Steinberg BA, Hellkamp AS, Lokhnygina Y, et al. Use and outcomes of antiarrhythmic therapy in patients with atrial fibrillation receiving oral anticoagulation: results from the ROCKET AF trial. Heart Rhythm. 2014;11(6):925–32. 24833235 10.1016/j.hrthm.2014.03.006 PMC4035424

[8] Ray WA, Chung CP, Stein CM, et al. Serious bleeding in patients with atrial fibrillation using diltiazem with apixaban or rivaroxaban. JAMA. 2024;331(18):1565–75. 38619832 10.1001/jama.2024.3867 PMC11019444

[9] Ray WA, Chung CP, Stein CM, et al. Risk for bleeding-related hospitalizations during use of amiodarone with apixaban or rivaroxaban in patients with atrial fibrillation: a retrospective cohort study. Ann Intern Med. 2023;176(6):769–78. 37216662 10.7326/M22-3238

[10] Hill K, Sucha E, Rhodes E, et al. Amiodarone, verapamil, or diltiazem use with direct oral anticoagulants and the risk of hemorrhage in older adults. CJC Open. 2022;4(3):315–23. 35386137 10.1016/j.cjco.2021.11.002 PMC8978070

[11] Gronich N, Stein N, Muszkat M. Association between use of pharmacokinetic-interacting drugs and effectiveness and safety of direct acting oral anticoagulants: nested case-control study. Clin Pharmacol Ther. 2021;110(6):1526–36. 34287842 10.1002/cpt.2369 PMC9290518

[12] Komatsu Y, Yodoshi M, Takegami M, Yokoyama S, Hosomi K. Association between hemorrhage and direct oral anticoagulants in combination with verapamil: analysis of Japanese Adverse Drug Event Report database and electronic medical record data. Int J Clin Pharmacol Ther. 2023;61(4):148–58. 36795612 10.5414/CP204310

[13] Wolf A, Dedman D, Campbell J, et al. Data resource profile: clinical practice research datalink (CPRD) aurum. Int J Epidemiol. 2019;48(6):1740–1740g. 30859197 10.1093/ije/dyz034 PMC6929522

[14] Yamreudeewong W, DeBisschop M, Martin LG, Lower DL. Potentially significant drug interactions of class III antiarrhythmic drugs. Drug Saf. 2003;26(6):421–38. 12688833 10.2165/00002018-200326060-00004

[15] Douros A, Cui Y, Platt RW, Filion KB, Sebastiani G, Renoux C. Effectiveness and safety of direct oral anticoagulants among patients with non-valvular atrial fibrillation and liver disease: a multinational cohort study. Thromb Res. 2024;237:71–8. 38552497 10.1016/j.thromres.2024.03.024

[16] Douros A, Renoux C, Coulombe J, Suissa S. Patterns of long-term use of non-vitamin K antagonist oral anticoagulants for non-valvular atrial fibrillation: Quebec observational study. Pharmacoepidem Dr S. 2017;26(12):1546–54. 10.1002/pds.4333 28984052

[17] Loo SY, Dell’Aniello S, Huiart L, Renoux C. Trends in the prescription of novel oral anticoagulants in UK primary care. Br J Clin Pharmacol. 2017;83(9):2096–106. 28390065 10.1111/bcp.13299 PMC5555878

[18] Mendell J, Zahir H, Matsushima N, et al. Drug-drug interaction studies of cardiovascular drugs involving P-glycoprotein, an efflux transporter, on the pharmacokinetics of Edoxaban, an oral factor Xa inhibitor. Am J Cardiovasc Drugs. 2013;13(5):331–42. 23784266 10.1007/s40256-013-0029-0 PMC3781304

[19] Ruff CT, Giugliano RP, Braunwald E, et al. Comparison of the efficacy and safety of new oral anticoagulants with warfarin in patients with atrial fibrillation: a meta-analysis of randomised trials. Lancet. 2014;383(9921):955–62. 24315724 10.1016/S0140-6736(13)62343-0

[20] Schmucker DL. Liver function and phase I drug metabolism in the elderly: a paradox. Drugs Aging. 2001;18(11):837–51. 11772124 10.2165/00002512-200118110-00005

[21] Miettinen OS. The need for randomization in the study of intended effects. Stat Med. 1983;2(2):267–71. 6648141 10.1002/sim.4780020222

[22] Dimakos J, Douros A. Methodological Considerations on the use of cohort designs in drug-drug interaction studies in pharmacoepidemiology. Curr Epidemiol Rep. 2024;11(3):175–83.

[23] Pham P, Schmidt S, Lesko L, Lip GYH, Brown JD. Association of oral anticoagulants and verapamil or diltiazem with adverse bleeding events in patients with nonvalvular atrial fibrillation and normal kidney function. JAMA Netw Open. 2020;3(4):e203593. 32329770 10.1001/jamanetworkopen.2020.3593 PMC7182798

