# Supplementary material for: Concomitant use of direct oral anticoagulants and interacting antiarrhythmic drugs and the risk of stroke and bleeding among patients with non-valvular atrial fibrillation: a multinational cohort study
Source: BMC Med. 2025 Oct 28;23:592. doi: 10.1186/s12916-025-04464-6 (PMC12570794; doi:10.1186/s12916-025-04464-6)
Supplement: Supplementary file 1 — Additional file 1: Figure S1. Study design. Figure S2. Flowchart illustrating the construction of the study cohort. Figure S3. Cumulative incidences of the study outcomes. Table S1. ICD-10 codes for the definition of ischemic stroke and major bleeding. Table S2. Baseline characteristics of patients in the CPRD. Table S3. Baseline characteristics of patients in the RAMQ. Table S4. Reasons for censoring. Table S5. Risk of ischemic stroke associated with concomitant use of DOACs and interacting antiarrhythmics compared with concomitant use of DOACs and non-interacting antiarrhythmics among patients with NVAF (stratification by demographics). Table S6. Risk of ischemic stroke associated with concomitant use of DOACs and interacting antiarrhythmics compared with concomitant use of DOACs and non-interacting antiarrhythmics (stratification by baseline risk, individual DOACs, and type of DOAC use). Table S7. Risk of major bleeding associated with concomitant use of DOACs and interacting antiarrhythmics compared with concomitant use of DOACs and non-interacting antiarrhythmics (stratification by demographics). Table S8. Risk of major bleeding associated with concomitant use of DOACs and interacting antiarrhythmics compared with concomitant use of DOACs and non-interacting antiarrhythmics (stratification by baseline risk, individual DOACs, and type of DOAC use). Table S9. Risk of ischemic stroke associated with concomitant use of DOACs and interacting antiarrhythmics compared with concomitant use of DOACs and non-interacting antiarrhythmics (sensitivity analyses). Table S10. Risk of major bleeding associated with concomitant use of DOACs and interacting antiarrhythmics compared with concomitant use of DOACs and non-interacting antiarrhythmics (sensitivity analyses). [file 12916_2025_4464_MOESM1_ESM.docx]

**Contents of Additional File 1**

[Figure S1. Study design 2](#_Toc211441815)

[Figure S2. Flowchart illustrating the construction of the study cohort 3](#_Toc211441816)

[Figure S3. Cumulative incidences of the study outcomes 4](#_Toc211441817)

[Table S1. ICD-10 codes for the definition of ischemic stroke and major bleeding 5](#_Toc211441818)

[Table S2. Baseline characteristics of patients in the CPRD 6](#_Toc211441819)

[Table S3. Baseline characteristics of patients in the RAMQ 9](#_Toc211441820)

[Table S4. Reasons for censoring 11](#_Toc211441821)

[Table S5. Risk of ischemic stroke associated with concomitant use of DOACs and interacting antiarrhythmics compared with concomitant use of DOACs and non-interacting antiarrhythmics among patients with NVAF (stratification by demographics) 12](#_Toc211441822)

[Table S6. Risk of ischemic stroke associated with concomitant use of DOACs and interacting antiarrhythmics compared with concomitant use of DOACs and non-interacting antiarrhythmics (stratification by baseline risk, individual DOACs, and type of DOAC use) 14](#_Toc211441823)

[Table S7. Risk of major bleeding associated with concomitant use of DOACs and interacting antiarrhythmics compared with concomitant use of DOACs and non-interacting antiarrhythmics (stratification by demographics) 16](#_Toc211441824)

[Table S8. Risk of major bleeding associated with concomitant use of DOACs and interacting antiarrhythmics compared with concomitant use of DOACs and non-interacting antiarrhythmics (stratification by baseline risk, individual DOACs, and type of DOAC use) 18](#_Toc211441825)

[Table S9. Risk of ischemic stroke associated with concomitant use of DOACs and interacting antiarrhythmics compared with concomitant use of DOACs and non-interacting antiarrhythmics (sensitivity analyses) 20](#_Toc211441826)

[Table S10. Risk of major bleeding associated with concomitant use of DOACs and interacting antiarrhythmics compared with concomitant use of DOACs and non-interacting antiarrhythmics (sensitivity analyses) 22](#_Toc211441827)

# **Figure S1. Study design**


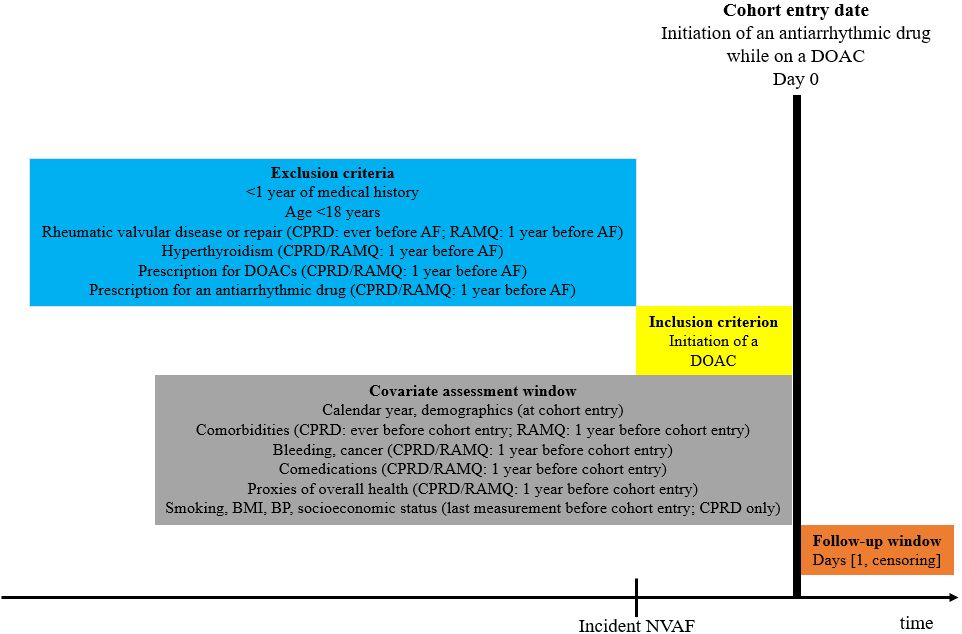


Abbreviations: CPRD, Clinical Practice Research Datalink; RAMQ, Régie de l'Assurance-Maladie du Québec; AF, atrial fibrillation; NVAF, non-valvular atrial fibrillation DOAC, direct oral anticoagulant; BMI, body mass index; BP, blood pressure.

# **Figure S2. Flowchart illustrating the construction of the study cohort**


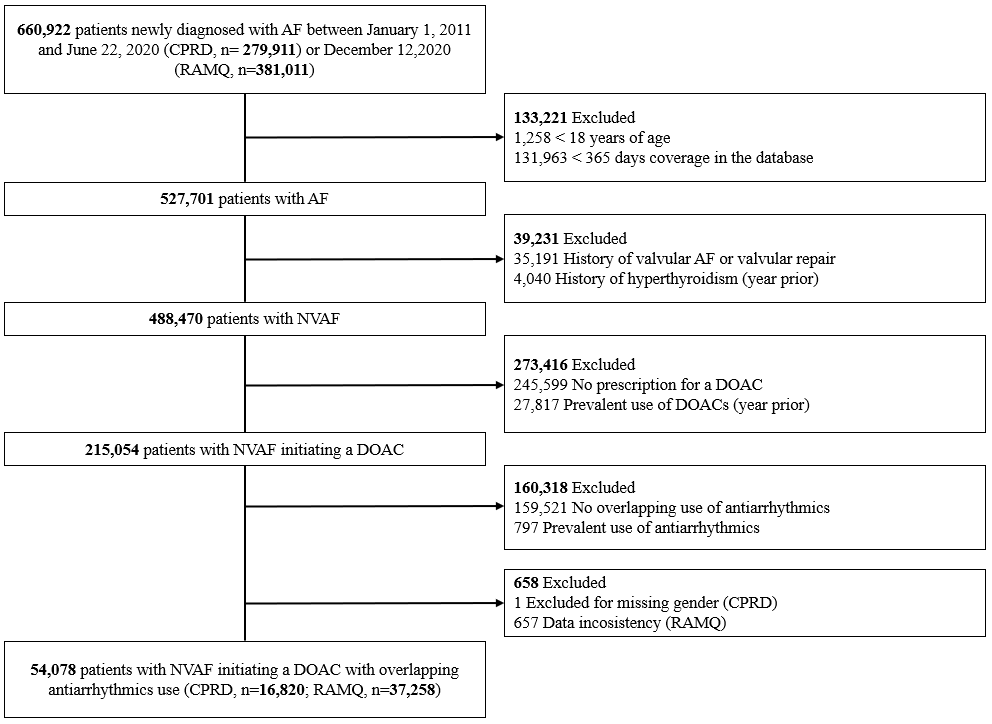


Abbreviations: AF, atrial fibrillation; CPRD, Clinical Practice Research Datalink; RAMQ, Régie de l'Assurance-Maladie du Québec; NVAF, non-valvular atrial fibrillation; DOAC, direct oral anticoagulant.

# **Figure S3. Cumulative incidences of the study outcomes**


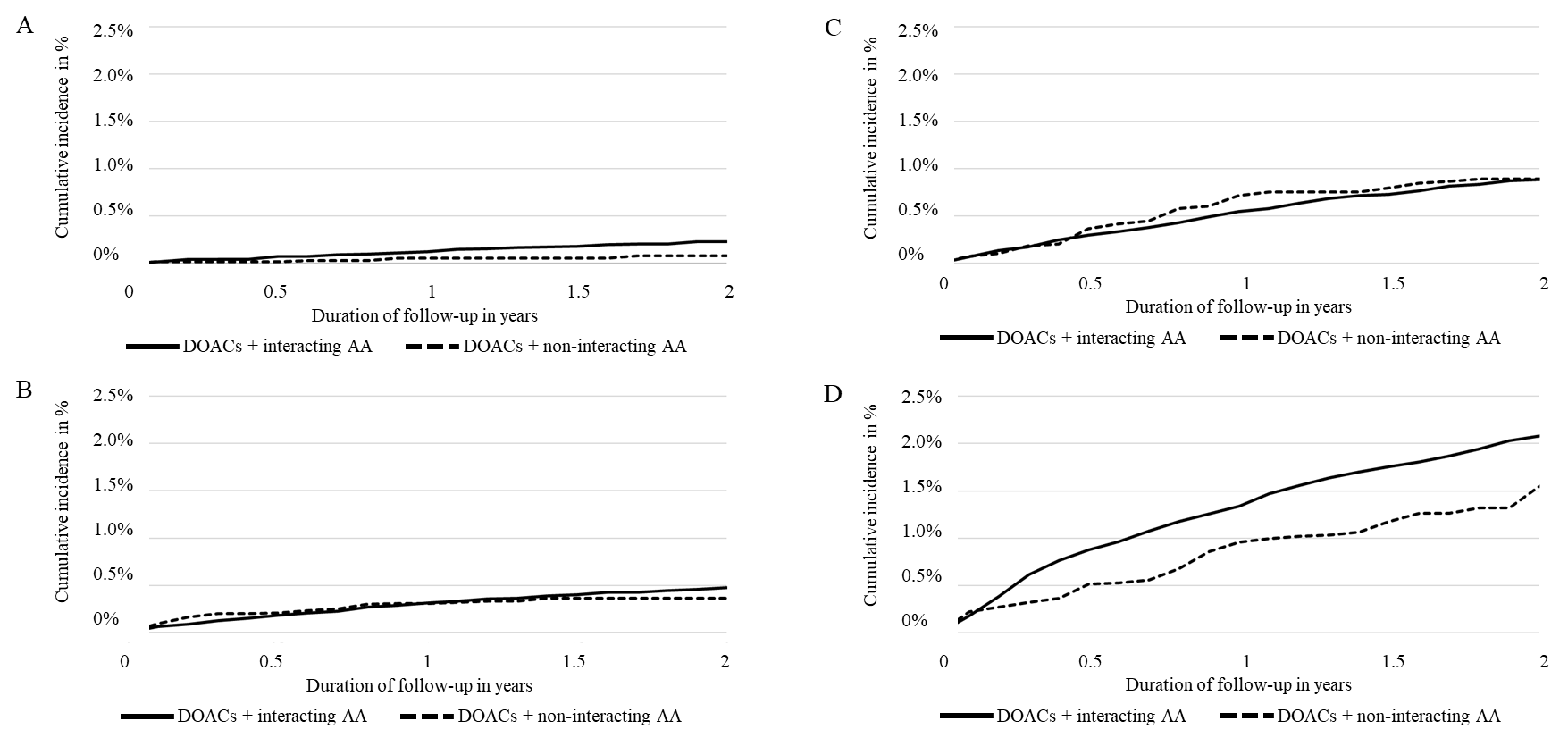


Panel A : 2-year cumulative incidence of ischemic stroke in the two exposure groups in the CPRD.

Panel B : 2-year cumulative incidence of ischemic stroke in the two exposure groups in the RAMQ.

Panel C : 2-year cumulative incidence of major bleeding in the two exposure groups in the CPRD.

Panel D : 2-year cumulative incidence of major bleeding in the two exposure groups in the RAMQ.

Abbreviations: CPRD, Clinical Practice Research Datalink; RAMQ, Régie de l'Assurance-Maladie du Québec; DOAC, direct oral anticoagulant; AA, antiarrhythmic drugs.

# **Table S1. ICD-10 codes for the definition of ischemic stroke and major bleeding**

| **Outcomes** | **ICD-10 code** |
| --- | --- |
| **Ischemic stroke / TIA / Systemic embolism** | I63, I64, H34.1, G45, H340, I74 |
| **Major bleeding (Intracranial bleeding)** | I60, I61, I621, I620, I629 |
| **Major bleeding (Gastrointestinal bleeding)** | I850, K920, K921, K922, K2211, K226, K228, K250, K252, K254, K256, K260, K262, K264, K266, K270, K272, K274, K276, K280, K282, K284, K286, K290, K294, K2921, K2961, K2971, K2991, K2981, K3181, K5711, K5713, K5731, K5733, K661, K625, K552 |
| **Major bleeding (Other major bleeding)** | D699, H0289, H0523, H113, H313, H356, H431, H44819, I230, I312, J94.2, M250, M7981, N02, N3289, N92, N950, N837, N939, R040, R041, R042, R048, R049, R233, R31, R58, T792, T810 |

Abbreviations: ICD-10, International Classification of diseases, 10^th^ Revision; TIA, transient ischemic attack.

**Table S2. Baseline characteristics of patients in the CPRD**

|  | **Before IPTW** |  | **SMD** | **After IPTW** |  | **SMD** |
| --- | --- | --- | --- | --- | --- | --- |
|  | **DOACs + interacting antiarrhythmics**  **(n=12,487)** | **DOACs + non interacting antiarrhythmics (n=4,333)** |  | **DOACs + interacting antiarrhythmics (n=12,477)** | **DOACs + non interacting antiarrhythmics (n=4,417)** |  |
| Age in years, mean (SD) | 72.45 (10.95) | 66.77 (11.10) | 0.515 | 71.01 (11.26) | 71.44 (11.39) | -0.038 |
| Female sex | 5,759 (46.12) | 2,185 (50.43) | -0.086 | 5,890 (47.21) | 2,082 (47.13) | 0.002 |
| Smoking status |  |  |  |  |  |  |
| Current | 1,104 (8.84) | 270 (6.23) | 0.099 | 1,017 (8.15) | 352 (7.98) | 0.006 |
| Former | 5,323 (42.63) | 1,514 (34.94) | 0.158 | 5,068 (40.62) | 1.817 (41.13) | -0.010 |
| Never | 6,040 (48.37) | 2,541 (58.64) | -0.207 | 6,373 (51.08) | 2,241 (50.73) | 0.007 |
| Unknown | 20 (0.16) | 8 (0.18) | -0.005 | 19 (0.15) | 7 (0.16) | -0.003 |
| Body mass index in kg/m^2^ |  |  |  |  |  |  |
| <25 | 2,765 (22.14) | 1,163 (26.84) | -0.109 | 2,909 (23.32) | 997 (22.57) | 0.018 |
| 25-29 | 4,292 (34.37) | 1,613 (37.23) | -0.060 | 4,385 (35.14) | 1,546 (34.99) | 0.003 |
| ≥30 | 5,121 (41.01) | 1,404 (32.40) | 0.179 | 4,849 (38.86) | 1,761 (39.88) | -0.021 |
| Unknown | 309 (2.47) | 153 (3.53) | -0.062 | 334 (2.68) | 113 (2.56) | 0.008 |
| Blood pressure control^*^ |  |  |  |  |  |  |
| Normal | 4,018 (32.18) | 1,356 (31.25) | 0.019 | 3,951 (31.67) | 1,319 (29.87) | 0.039 |
| High | 8,460 (67.75) | 2,969 (68.52) | -0.017 | 8,516 (68.25) | 3,091 (69.98) | -0.037 |
| Unknown | 9 (0.07) | 8 (0.18) | -0.031 | 10 (0.08) | 7 (0.15) | -0.021 |
| **Comorbidities** |  |  |  |  |  |  |
| Alcohol-related disorders | 3,917 (31.37) | 1,151 (26.56) | 0.106 | 3,744 (30.01) | 1,317 (29.82) | 0.004 |
| Arterial hypertension | 9,172 (73.45) | 2,473 (57.07) | 0.349 | 8,655 (69.37) | 3,127 (70.80) | -0.031 |
| Prior ischemic stroke/TIA | 1,854 (14.85) | 480 (11.08) | 0.112 | 1,729 (13.86) | 591 (13.38) | 0.014 |
| Congestive heart failure | 3,942 (31.57) | 559 (12.90) | 0.461 | 3,350 (26.85) | 1,277 (28.91) | -0.046 |
| Coronary artery disease | 4,698 (37.62) | 986 (22.76) | 0.328 | 4,228 (33.89) | 1,574 (35.63) | -0.037 |
| Peripheral vascular disease | 1,214 (9.72) | 173 (3.99) | 0.228 | 1,032 (8.28) | 436 (9,87) | -0.055 |
| Prior major bleeding | 465 (3.72) | 102 (2.35) | 0.080 | 422 (3.38) | 153 (3.47) | -0.005 |
| Diabetes mellitus | 3,492 (27.97) | 794 (18.32) | 0.230 | 3,174 (25.44) | 1,094 (24.77) | 0.015 |
| Liver disease | 592 (4.74) | 163 (3.76) | 0.049 | 558 (4.47) | 177 (4.00) | 0.023 |
| Renal disease | 6,169 (49.40) | 1,399 (32.29) | 0.353 | 5,624 (45.07) | 2,022 (45.77) | -0.014 |
| Cancer | 660 (5.29) | 174 (4.02) | 0.060 | 617 (4.95) | 220 (4.98) | -0.001 |
| **Comedications** |  |  |  |  |  |  |
| Antiplatelet agents | 4,436 (35.52) | 1,154 (26.63) | 0.193 | 4,161 (33.35) | 1,500 (33.96) | -0.013 |
| Non-steroidal anti-inflammatory drugs | 1,347 (10.79) | 492 (11.35) | -0.018 | 1,350 (10.82) | 479 (10.84) | -0.001 |
| Selective serotonin reuptake inhibitors | 1.322 (10.59) | 367 (8.47) | 0.072 | 1,250 (10.02) | 440 (9.96) | 0.002 |
| Proton pump inhibitors | 5,930 (47.49) | 1,785 (41.20) | 0.127 | 5,727 (45.90) | 2,047 (46.34) | -0.009 |
| H_2_ blockers | 650 (5.21) | 168 (3.88) | 0.064 | 610 (4.89) | 221 (5.01) | -0.006 |
| Vitamin K antagonists | 2.192 (17.55) | 645 (14.89) | 0.072 | 2,177 (16.97) | 805 (18.23) | -0.033 |
| N Hospitalizations in the past year |  |  |  |  |  |  |
| 0 | 8,086 (64.76) | 3,255 (75.12) | -0.227 | 8,407 (67.38) | 2,984 (67.55) | -0.004 |
| 1 | 2,697 (21.60) | 733 (16.92) | 0.119 | 2,541 (20.37) | 867 (19.63) | 0.019 |
| ≥2 | 1,704 (13.65) | 345 (7.96) | 0.184 | 1,529 (12.25) | 566 (12.82) | -0.017 |
| Index of multiple deprivation |  |  |  |  |  |  |
| 1 | 1,809 (14.49) | 773 (17.84) | 0.091 | 1,922 (15.41) | 677 (15.33) | 0.002 |
| 2 | 1,682 (13.47) | 656 (15.14) | -0.048 | 1,740 (13.94) | 642 (14.54) | -0.017 |
| 3 | 1,737 (13.91) | 608 (14.03) | -0.003 | 1,750 (14.02) | 651 (14.73) | -0.020 |
| 4 | 1,934 (15.49) | 552 (12.74) | 0.079 | 1,840 (14.75) | 633 (14.33) | 0.012 |
| 5 | 1,542 (12.35) | 347 (8.01) | 0.144 | 1,396 (11.19) | 477 (10.80) | 0.012 |
| Unknown | 3,783 (30.30) | 1,397 (32.24) | -0.042 | 3,829 (30.69) | 1,337 (30.27) | 0.009 |
| Time since NVAF diagnosis in months |  |  |  |  |  |  |
| 0-3 | 4,807 (38.50) | 1.087 (25.09) | 0.291 | 4,365 (34.98) | 1,541 (34.88) | 0.002 |
| 4-6 | 1,553 (12.44) | 535 (12.35) | 0.003 | 1,550 (12.43) | 534 (12.10) | 0.010 |
| >6 | 6,127 (49.07) | 2,711 (62.57) | 0.274 | 6,562 (52.59) | 2,342 (53.02) | 0.009 |
| DOACs |  |  |  |  |  |  |
| Apixaban | 5,632 (45.10) | 1,738 (40.11) |  |  |  |  |
| Dabigatran | 1,122 (8.99) | 457 (10.55) |  |  |  |  |
| Edoxaban | 428 (3.43) | 154 (3.55) |  |  |  |  |
| Rivaroxaban | 5,304 (42.48) | 1,983 (45.77) |  |  |  |  |
| Interacting antiarrhythmic drugs |  |  |  |  |  |  |
| Amiodarone | 5,261 (42.13) | 0 |  |  |  |  |
| Diltiazem | 5,536 (44.33) | 0 |  |  |  |  |
| Dronedarone | 176 (1.41) | 0 |  |  |  |  |
| Verapamil | 1,455 (11.65) | 0 |  |  |  |  |
| Non-interacting antiarrhythmic drugs |  |  |  |  |  |  |
| Flecainide | 0 | 2,596 (59.91) |  |  |  |  |
| Propafenone | 0 | 68 (1.57) |  |  |  |  |
| Sotalol | 0 | 1,667 (38.47) |  |  |  |  |
| CHA₂DS₂-VASc score, mean (SD) | 3.74 (1.85) | 2.72 (1.73) |  | 3.48 (1.87) | 3.55 (1.89) |  |
| HAS-BLED score, mean (SD) | 2.90 (1.38) | 2.23 (1.36) |  | 2.73 (1.41) | 2.73 (1.37) |  |
| eGFR, mean (SD) | 67.12 (17.02) | 73.94 (14.65) |  | 68.43 (16.76) | 69.94 (16.43) |  |

All values are n (%) unless indicated otherwise.

^*^ Normal blood pressure control was defined as systolic blood pressure <130 mmHg and diastolic blood pressure <80 mmHg, while high blood pressure was defined as systolic blood pressure ≥130 mmHg or diastolic blood pressure ≥80 mmHg.

Abbreviations: CPRD, Clinical Practice Research Datalink; IPTW, inverse probability treatment weighting; SD, standard deviation; CHA_2_DS_2_-VASc, congestive heart failure, hypertension, age ≥75 years, diabetes mellitus, stroke, vascular disease, age 65-74 years, sex; HAS-BLED, hypertension, abnormal renal or liver function, stroke, bleeding, elderly, drugs or excess alcohol use; eGFR, estimated glomerular filtration rate; SMD, standardized mean difference; DOACs, direct oral anticoagulants; TIA, transient ischemic attack; NVAF, non-valvular atrial fibrillation; CHA₂DS₂-VASc, congestive heart failure, hypertension, age ≥75 years, diabetes mellitus, stroke, vascular disease, age 65-74 years, sex; HAS-BLED, hypertension, abnormal renal or liver function, stroke, bleeding, elderly, drugs or excess alcohol use; eGFR, estimated glomerular filtration rate.

**Table S3. Baseline characteristics of patients in the RAMQ**

|  | **Before IPTW** |  | **SMD** | **After IPTW** |  | **SMD** |
| --- | --- | --- | --- | --- | --- | --- |
|  | **DOACs + interacting antiarrhythmics (n=28,504)** | **DOACs + non interacting antiarrhythmics (8,754)** |  | **DOACs + interacting antiarrhythmics (n=28,483)** | **DOACs + non interacting antiarrhythmics (n=8,915)** |  |
| Age in years, mean (SD) | 75.95 (9.36) | 70.84 (9.00) | 0.557 | 74.77 (9.51) | 75.05 (9.61) | -0.029 |
| Female sex (%) | 15,452 (53.86) | 4,635 (52.95) | 0.018 | 15,267 (53.60) | 4,711 (52.84) | 0.015 |
| **Comorbidities** |  |  |  |  |  |  |
| Alcohol-related disorders | 1,730 (6.07) | 233 (2.66) | 0.167 | 1,502 (5.27) | 553 (6.21) | -0.040 |
| Arterial hypertension | 25,896 (90.85) | 7,906 (90.31) | 0.018 | 25,835 (90.71) | 8,057 (90.38) | 0.011 |
| Prior ischemic stroke/TIA | 2,539 (8.91) | 612 (6.99) | 0.071 | 2,418 (8.49) | 806 (9.04) | -0.019 |
| Congestive heart failure | 10,614 (37.24) | 1,259 (14.38) | 0.541 | 9,083 (31.89) | 2,921 (32.76) | -0.019 |
| Coronary artery disease | 15,295 (53.66) | 3,576 (40.85) | 0.259 | 14,426 (50.65) | 4,496 (50.43) | 0.004 |
| Peripheral vascular disease | 4,566 (16.02) | 661 (7.55) | 0.265 | 4,002 (14.05) | 1,299 (14.57) | -0.015 |
| Prior major bleeding | 4,044 (14.19) | 835 (9.54) | 0.144 | 3,728 (13.09) | 1,198 (13.44) | -0.010 |
| Diabetes mellitus | 9,467 (33.21) | 2,110 (24.10) | 0.203 | 8,877 (31.17) | 2,905 (32.58) | -0.030 |
| Liver disease | 1,917 (6.73) | 354 (4.04) | 0.119 | 1,737 (6.10) | 593 (6.65) | -0.023 |
| Renal disease | 5,832 (20.46) | 670 (7.65) | 0.375 | 4,977 (17.47) | 1,664 (18.67) | -0.031 |
| Cancer | 5,221 (18.32) | 1,305 (14.91) | 0.092 | 4,996 (17.54) | 1,637 (18.37) | -0.022 |
| **Comedications** |  |  |  |  |  |  |
| Antiplatelet agents | 13,291 (46.63) | 3,589 (41.00) | 0.114 | 12,903 (45.30) | 4,038 (45.30) | <0.001 |
| Non-steroidal anti-inflammatory drugs | 3,844 (13.49) | 1,435 (16.39) | -0.081 | 4,028 (14.14) | 1,227 (13.76) | 0.011 |
| Selective serotonin reuptake inhibitors | 3,332 (11.69) | 761 (8.69) | 0.099 | 3,131 (10.99) | 955 (10.72) | 0.009 |
| Proton pump inhibitors | 15,740 (55.22) | 4,196 (47.93) | 0.146 | 15,253 (53.55) | 4,824 (54.11) | -0.011 |
| H_2_ blockers | 727 (2.55) | 192 (2.19) | 0.024 | 702 (2.46) | 221 (2.48) | -0.001 |
| Vitamin K antagonists | 5,704 (20.01) | 1,318 (15.06) | 0.130 | 5.397 (18.95) | 1,749 (19.62) | -0.017 |
| N Hospitalizations in the past year |  |  |  |  |  |  |
| 0 | 9,131 (32.03) | 4,838 (55.27) | -0.482 | 10,698 (37.56) | 3,369 (37.79) | -0.005 |
| 1 | 12,043 (42.25) | 2,872 (32.81) | 0.196 | 11,376 (39.94) | 3,450 (38.70) | 0.025 |
| ≥2 | 7,330 (25.72) | 1,044 (11.93) | 0.358 | 6,409 (22.50) | 2,096 (23.51) | -0.024 |
| Time since NVAF diagnosis in months |  |  |  |  |  |  |
| 0-1 | 9,948 (34.90) | 2,696 (30.80) | 0.087 | 9,670 (33.95) | 3,036 (34.06) | -0.002 |
| 1-3 | 3,005 (10.54) | 993 (11.34) | -0.026 | 3,070 (10.78) | 991 (11.11) | -0.011 |
| >3 | 15,551 (54-56) | 5,065 (57.86) | -0.067 | 15,743 (55.27) | 4,888 (54.83) | 0.009 |
| DOACs |  |  |  |  |  |  |
| Apixaban | 14,903 (52.28) | 3,876 (44.28) |  |  |  |  |
| Dabigatran | 3,654 (12.82) | 1,315 (15.02) |  |  |  |  |
| Edoxaban | 379 (1.33) | 166 (1,90) |  |  |  |  |
| Rivaroxaban | 9,564 (33.55) | 3,395 (38.78) |  |  |  |  |
| Interacting antiarrhythmic drugs |  |  |  |  |  |  |
| Amiodarone | 11,184 (39.24) | 0 |  |  |  |  |
| Diltiazem | 15,895 (55.76) | 0 |  |  |  |  |
| Dronedarone | 7 (0.02) | 0 |  |  |  |  |
| Verapamil | 887 (3.11) | 0 |  |  |  |  |
| Non-interacting antiarrhythmic drugs |  |  |  |  |  |  |
| Flecainide | 0 | 2,792 (31.89) |  |  |  |  |
| Propafenone | 0 | 1,702 (19.44) |  |  |  |  |
| Sotalol | 0 | 4,253 (48.58) |  |  |  |  |
| CHA₂DS₂-VASc score, mean (SD) | 4.38 (1.53) | 3.54 (1.45) |  | 4.23 (1.69) | 4.18 (1.55) |  |
| HAS-BLED score, mean (SD) | 1.98 (1.00) | 1.60 (0.90) |  | 1.90 (0.99) | 1.94 (1.03) |  |

All values are n (%) unless indicated otherwise.

Abbreviations: RAMQ, Régie de l'Assurance-Maladie du Québec; IPTW, inverse probability treatment weighting; SMD, standardized mean difference; SD, standard deviation; CHA_2_DS_2_-VASc, congestive heart failure, hypertension, age ≥75 years, diabetes mellitus, stroke, vascular disease, age 65-74 years, sex; HAS-BLED, hypertension, abnormal renal or liver function, stroke, bleeding, elderly, drugs or excess alcohol use; DOACs, direct oral anticoagulants; TIA, transient ischemic attack.

**Table S4. Reasons for censoring**

|  | **N**  **Patients** | **Treatment discontinuation (%)** | **Treatment switch (%)** | **Administrative censoring (%)** | **Death (%)** | **Outcome (%)** |
| --- | --- | --- | --- | --- | --- | --- |
| **Ischemic stroke / TIA / SE** |  |  |  |  |  |  |
| **CPRD** |  |  |  |  |  |  |
| DOACs + interacting antiarrhythmics | 12,487 | 8,335 (66.75) | 224 (1.79) | 3,273 (26.21) | 563 (4.51) | 92 (0.74) |
| DOACs + non-interacting antiarrhythmics | 4,333 | 2,927 (67.55) | 200 (4.62) | 1,146 (26.45) | 44 (1.02) | 16 (0.37) |
| **RAMQ** |  |  |  |  |  |  |
| DOACs + interacting antiarrhythmics | 28,504 | 14,843 (62.07) | 1,167 (4.09) | 10,324 (36.22) | 1,554 (5.45) | 616 (2.16) |
| DOACs + non-interacting antiarrhythmics | 8,754 | 4,375 (49.98) | 1,296 (14.80) | 2,825 (32.27) | 135 (1.54) | 123 (1.41) |
| **Major bleeding** |  |  |  |  |  |  |
| **CPRD** |  |  |  |  |  |  |
| DOACs + interacting antiarrhythmics | 12,487 | 8,142 (65.20) | 221 (1.77) | 3,189 (25.54) | 527 (4.22) | 408 (3.27) |
| DOACs + non-interacting antiarrhythmics | 4,333 | 2,882 (66.51) | 200 (4.62) | 1,119 (25.83) | 40 (0.92) | 92 (2.12) |
| **RAMQ** |  |  |  |  |  |  |
| DOACs + interacting antiarrhythmics | 28,504 | 13,839 (48.55) | 1,146 (4.02) | 9,758 (34.23) | 1,388 (4.87) | 2,373 (8.33) |
| DOACs + non-interacting antiarrhythmics | 8,754 | 4,234 (48.37) | 1,263 (14.43) | 2,751 (31.43) | 131 (1.50) | 375 (4.28) |

Abbreviations: CPRD, Clinical Practice Research Datalink; RAMQ, Régie de l'Assurance-Maladie du Québec; DOACs, direct oral anticoagulants; TIA, transient ischemic attack; SE, systemic embolism.

**Table S5. Risk of ischemic stroke associated with concomitant use of DOACs and interacting antiarrhythmics compared with concomitant use of DOACs and non-interacting antiarrhythmics among patients with NVAF (stratification by demographics)**

|  | **N**  **Patients** | **N**  **Events** | **N**  **PY** | | **IR^*^** | **Crude HR**  **(95%CI)** | **IPTW HR**  **(95%CI)** | **Pooled HR**  **(95% CI)** | **I^2^** |
| --- | --- | --- | --- | --- | --- | --- | --- | --- | --- |
| **<70 years** |  |  |  |  | |  |  | 1.00 (0.73-1.36) | 0% |
| **CPRD** |  |  |  |  | |  |  |  |  |
| DOACs + interacting antiarrhythmics | 4,485 | 24 | 3,454 | 6.95 | | 1.38 (0.64-2.96) | 1.12 (0.55-2.30) |  |  |
| DOACs + non interacting antiarrhythmics | 2,514 | 9 | 1,801 | 5,00 | | 1.00 (reference) | 1.00 (reference) |  |  |
| **RAMQ** |  |  |  |  | |  |  |  |  |
| DOACs + interacting antiarrhythmics | 6,687 | 101 | 10,137 | 9,96 | | 1.72 (1.16-2.55) | 0.97 (0.69-1.37) |  |  |
| DOACs + non interacting antiarrhythmics | 3,648 | 33 | 5,756 | 5.73 | | 1.00 (reference) | 1.00 (reference) |  |  |
| **≥70 years** |  |  |  |  | |  |  | 1.05 (0.87-1.28) | 0% |
| **CPRD** |  |  |  |  | |  |  |  |  |
| DOACs + interacting antiarrhythmics | 8,002 | 68 | 6,665 | 10.20 | | 2.39 (1.10-5.21) | 1.10 (0.62-1.96) |  |  |
| DOACs + non interacting antiarrhythmics | 1,819 | 7 | 1,683 | 4.16 | | 1.00 (reference) | 1.00 (reference) |  |  |
| **RAMQ** |  |  |  |  | |  |  |  |  |
| DOACs + interacting antiarrhythmics | 21,817 | 515 | 33,847 | 15.22 | | 1.40 (1.12-1.75) | 1.05 (0.85-1.29) |  |  |
| DOACs + non interacting antiarrhythmics | 5,106 | 90 | 8,416 | 10.69 | | 1.00 (reference) | 1.00 (reference) |  |  |
| **Female sex** |  |  |  |  | |  |  | 1.01 (0.81-1.25) | 84% |
| **CPRD** |  |  |  |  | |  |  |  |  |
| DOACs + interacting antiarrhythmics | 5,759 | 46 | 4,908 | 9.37 | | 2.89 (1.23-6.77) | 3.28 (1.28-8.42) |  |  |
| DOACs + non interacting antiarrhythmics | 2,185 | 6 | 1,855 | 3,23 | | 1.00 (reference) | 1.00 (reference) |  |  |
| **RAMQ** |  |  |  |  | |  |  |  |  |
| DOACs + interacting antiarrhythmics | 15,352 | 354 | 24,761 | 14.30 | | 1.58 (1.22-2.04) | 0.95 (0.76-1.18) |  |  |
| DOACs + non interacting antiarrhythmics | 4,635 | 69 | 7,690 | 8.97 | | 1.00 (reference) | 1.00 (reference) |  |  |
| **Male sex** |  |  |  |  | |  |  | 0.95 (0.75-1.20) | 81% |
| **CPRD** |  |  |  |  | |  |  |  |  |
| DOACs + interacting antiarrhythmics | 6,728 | 46 | 5,211 | 8.83 | | 1.42 (0.72-2.81) | 0.58 (0.36-0.94) |  |  |
| DOACs + non interacting antiarrhythmics | 2,148 | 10 | 1,629 | 6.14 | | 1.00 (reference) | 1.00 (reference) |  |  |
| **RAMQ** |  |  |  |  | |  |  |  |  |
| DOACs + interacting antiarrhythmics | 13,152 | 262 | 19,223 | 13.63 | | 1.61 (1.20-2.16) | 1.10 (0.85-1.44) |  |  |
| DOACs + non interacting antiarrhythmics | 4,119 | 54 | 6,481 | 8.33 | | 1.00 (reference) | 1.00 (reference) |  |  |

^*^ IR per 1,000 PY.

Abbreviations: DOACs, direct oral anticoagulants; NVAF, non-valvular atrial fibrillation; PY, patient years; IR, incidence rate; HR, hazard ratio; CI, confidence interval; IPTW, inverse probability of treatment weighting; CPRD, Clinical Practice Research Datalink; RAMQ, Régie de l'Assurance-Maladie du Québec.

**Table S6. Risk of ischemic stroke associated with concomitant use of DOACs and interacting antiarrhythmics compared with concomitant use of DOACs and non-interacting antiarrhythmics (stratification by baseline risk, individual DOACs, and type of DOAC use)**

|  | **N**  **Patients** | **N**  **Events** | **N**  **PY** | **IR^*^** | **Crude HR**  **(95%CI)** | **IPTW HR**  **(95%CI)** | **Pooled HR**  **(95% CI)** | **I^2^** |
| --- | --- | --- | --- | --- | --- | --- | --- | --- |
| **CHA_2_DS_2_-VASC 0-4** |  |  |  |  |  |  | 0.92 (0.74-1.16) | 0% |
| **CPRD** |  |  |  |  |  |  |  |  |
| DOACs + interacting antiarrhythmics | 8,275 | 34 | 6,583 | 5.16 | 1.33 (0.68-2.63) | 0.47 (0.29-0.76) |  |  |
| DOACs + non interacting antiarrhythmics | 3,659 | 11 | 2,864 | 3.84 | 1.00 (reference) | 1.00 (reference) |  |  |
| **RAMQ** |  |  |  |  |  |  |  |  |
| DOACs + interacting antiarrhythmics | 15,231 | 219 | 24,747 | 8.85 | 1.55 (1.17-2.05) | 1.13 (0.87-1.46) |  |  |
| DOACs + non interacting antiarrhythmics | 6,646 | 62 | 10,896 | 5.69 | 1.00 (reference) | 1.00 (reference) |  |  |
| **CHA_2_DS_2_-VASC ≥5** |  |  |  |  |  |  | 0.98 (0.76-1.26) | 68% |
| **CPRD** |  |  |  |  |  |  |  |  |
| DOACs + interacting antiarrhythmics | 4,212 | 58 | 3,535 | 16.41 | 1.98 (0.79-4.93) | 2.37 (0.86-6.49) |  |  |
| DOACs + non interacting antiarrhythmics | 674 | 5 | 620 | 8.06 | 1.00 (reference) | 1.00 (reference) |  |  |
| **RAMQ** |  |  |  |  |  |  |  |  |
| DOACs + interacting antiarrhythmics | 13,273 | 397 | 19,237 | 20.64 | 1.09 (0.83-1.42) | 0.93 (0.71-1.20) |  |  |
| DOACs + non interacting antiarrhythmics | 2,108 | 61 | 3,276 | 18.62 | 1.00 (reference) | 1.00 (reference) |  |  |
| **Apixaban** |  |  |  |  |  |  | 0.99 (0.78-1.26) | 46% |
| **CPRD** |  |  |  |  |  |  |  |  |
| Apixaban + interacting antiarrhythmics | 5,632 | 42 | 4,146 | 10.13 | 1.88 (0.85-4.19) | 1.61 (0.77-3.37) |  |  |
| Apixaban + no inhibitors | 1,738 | 7 | 1,309 | 5.35 | 1.00 (reference) | 1.00 (reference) |  |  |
| **RAMQ** |  |  |  |  |  |  |  |  |
| Apixaban + interacting antiarrhythmics | 14,903 | 289 | 19,690 | 14.68 | 1.43 (1.07-1.90) | 0.93 (0.73-1.20) |  |  |
| Apixaban + non interacting antiarrhythmics | 3,876 | 56 | 5,615 | 9.97 | 1.00 (reference) | 1.00 (reference) |  |  |
| **Rivaroxaban** |  |  |  |  |  |  | 0.91 (0.70-1.19) | 69% |
| **CPRD** |  |  |  |  |  |  |  |  |
| Rivaroxaban + interacting antiarrhythmics | 5,304 | 35 | 4,481 | 7.81 | 1.55 (0.72-3,35) | 0.58 (0.33-1.01) |  |  |
| Rivaroxaban + non interacting antiarrhythmics | 1,983 | 8 | 1,586 | 5.04 | 1.00 (reference) | 1.00 (reference) |  |  |
| **RAMQ** |  |  |  |  |  |  |  |  |
| Rivaroxaban + interacting antiarrhythmics | 9,564 | 183 | 14,656 | 12.49 | 1.68 (1.19-2.36) | 1.04 (0.77-1.39) |  |  |
| Rivaroxaban + non interacting antiarrhythmics | 3,395 | 40 | 5,424 | 7.37 | 1.00 (reference) | 1.00 (reference) |  |  |
| **New DOAC use** |  |  |  |  |  |  | 1.15 (1.01-1.31) | 80% |
| **CPRD** |  |  |  |  |  |  |  |  |
| DOACs + interacting antiarrhythmics | 4,006 | 29 | 3,350 | 8.66 | 1.82 (0.70-4.70) | 0.59 (0.32-1.08) |  |  |
| DOACs + non interacting antiarrhythmics | 1,238 | 5 | 1,062 | 4.71 | 1.00 (reference) | 1.00 (reference) |  |  |
| **RAMQ** |  |  |  |  |  |  |  |  |
| DOACs + interacting antiarrhythmics | 15,550 | 329 | 23,465 | 14.02 | 1.48 (1.13-1.95) | 1.10 (0.85-1.42) |  |  |
| DOACs + non interacting antiarrhythmics | 4,049 | 61 | 6,606 | 9.23 | 1.00 (reference) | 1.00 (reference) |  |  |
| **Prevalent DOAC use** |  |  |  |  |  |  | 1.05 (0.84-1.30) | 63%l |
| **CPRD** |  |  |  |  |  |  |  |  |
| DOACs + interacting antiarrhythmics | 8,481 | 63 | 6,769 | 9.31 | 2.03 (1.07-3.85) | 1.68 (0.92-3.07) |  |  |
| DOACs + non interacting antiarrhythmics | 3,095 | 11 | 2,422 | 4.54 | 1.00 (reference) | 1.00 (reference) |  |  |
| **RAMQ** |  |  |  |  |  |  |  |  |
| DOACs + interacting antiarrhythmics | 12,954 | 287 | 20,518 | 13,99 | 1.70 (1.29-2.23) | 0.97 (0.77-1.23) |  |  |
| DOACs + non interacting antiarrhythmics | 4,705 | 62 | 7,566 | 8,19 | 1.00 (reference) | 1.00 (reference) |  |  |

^*^ IR per 1,000 PY.

Abbreviations: DOACs, direct oral anticoagulants; NVAF, non-valvular atrial fibrillation; CHA_2_DS_2_-VASc, congestive heart failure, hypertension, age ≥75 years, diabetes mellitus, stroke, vascular disease, age 65-74 years, sex; PY, patient years; IR, incidence rate; HR, hazard ratio; CI, confidence interval; IPTW, inverse probability of treatment weighting; CPRD, Clinical Practice Research Datalink; RAMQ, Régie de l'Assurance-Maladie du Québec.

**Table S7. Risk of major bleeding associated with concomitant use of DOACs and interacting antiarrhythmics compared with concomitant use of DOACs and non-interacting antiarrhythmics (stratification by demographics)**

|  | **N**  **Patients** | **N**  **Events** | **N**  **PY** | **IR^*^** | **Crude HR**  **(95%CI)** | **IPTW HR**  **(95%CI)** | **Pooled HR**  **(95% CI)** | **I^2^** |
| --- | --- | --- | --- | --- | --- | --- | --- | --- |
| **<70 years** |  |  |  |  |  |  | 1.56 (1.31-1.86) | 0% |
| **CPRD** |  |  |  |  |  |  |  |  |
| DOACs + interacting antiarrhythmics | 4,485 | 104 | 3,394 | 30.65 | 1.62 (1.10-2.39) | 1.43 (0.99-2.06) |  |  |
| DOACs + non interacting antiarrhythmics | 2,514 | 34 | 1,782 | 19.08 | 1.00 (reference) | 1.00 (reference) |  |  |
| **RAMQ** |  |  |  |  |  |  |  |  |
| DOACs + interacting antiarrhythmics | 6,687 | 415 | 9,816 | 42.28 | 2.25 (1.82-2.79) | 1.61 (1.32-1.96) |  |  |
| DOACs + non interacting antiarrhythmics | 3,648 | 105 | 5,690 | 18.45 | 1.00 (reference) | 1.00 (reference) |  |  |
| **≥70 years** |  |  |  |  |  |  | 1.26 (1.13-1.40) | 44% |
| **CPRD** |  |  |  |  |  |  |  |  |
| DOACs + interacting antiarrhythmics | 8,002 | 304 | 6,537 | 46.50 | 1.30 (0.98-1.72) | 1.07 (0.82-1.39) |  |  |
| DOACs + non interacting antiarrhythmics | 1,819 | 58 | 1,648 | 35,19 | 1.00 (reference) | 1.00 (reference) |  |  |
| **RAMQ** |  |  |  |  |  |  |  |  |
| DOACs + interacting antiarrhythmics | 21,817 | 1,958 | 32,661 | 59.95 | 1.79 (1.58-2.03) | 1.30 (1.16-1.46) |  |  |
| DOACs + non interacting antiarrhythmics | 5,106 | 270 | 8,259 | 32.69 | 1.00 (reference) | 1.00 (reference) |  |  |
| **Female sex** |  |  |  |  |  |  | 1.32 (1.17-1.50) | 4% |
| **CPRD** |  |  |  |  |  |  |  |  |
| DOACs + interacting antiarrhythmics | 5,759 | 205 | 4,798 | 42.72 | 1.50 (1.10-2.03) | 1.16 (0.88-1.54) |  |  |
| DOACs + non interacting antiarrhythmics | 2,185 | 52 | 1,823 | 28.52 | 1.00 (reference) | 1.00 (reference) |  |  |
| **RAMQ** |  |  |  |  |  |  |  |  |
| DOACs + interacting antiarrhythmics | 15,352 | 1,193 | 23,946 | 49.82 | 1.95 (1.67-2.27) | 1.37 (1.19-1.57) |  |  |
| DOACs + non interacting antiarrhythmics | 4,635 | 190 | 7,563 | 25.12 | 1.00 (reference) | 1.00 (reference) |  |  |
| **Male sex** |  |  |  |  |  |  | 1.32 (1.16-1.49) | 0% |
| **CPRD** |  |  |  |  |  |  |  |  |
| DOACs + interacting antiarrhythmics | 6,728 | 203 | 5,133 | 39.55 | 1.58 (1.13-2.22) | 1.18 (0.88-1.59) |  |  |
| DOACs + non interacting antiarrhythmics | 2,148 | 40 | 1,607 | 24.89 | 1.00 (reference) | 1.00 (reference) |  |  |
| **RAMQ** |  |  |  |  |  |  |  |  |
| DOACs + interacting antiarrhythmics | 13,152 | 1,180 | 18,530 | 63.68 | 2.13 (1.83-2.49) | 1.35 (1.18-1.54) |  |  |
| DOACs + non interacting antiarrhythmics | 4,119 | 185 | 6,386 | 28.97 | 1.00 (reference) | 1.00 (reference) |  |  |

^*^ IR per 1,000 PY.

Abbreviations: DOACs, direct oral anticoagulants; NVAF, non-valvular atrial fibrillation; PY, patient years; IR, incidence rate; HR, hazard ratio; CI, confidence interval; IPTW, inverse probability of treatment weighting; CPRD, Clinical Practice Research Datalink; RAMQ, Régie de l'Assurance-Maladie du Québec.

**Table S8. Risk of major bleeding associated with concomitant use of DOACs and interacting antiarrhythmics compared with concomitant use of DOACs and non-interacting antiarrhythmics (stratification by baseline risk, individual DOACs, and type of DOAC use)**

|  | **N**  **Patients** | **N**  **Events** | **N**  **PY** | **IR^*^** | **Crude HR**  **(95%CI)** | **IPTW HR**  **(95%CI)** | **Pooled HR**  **(95% CI)** | **I^2^** |
| --- | --- | --- | --- | --- | --- | --- | --- | --- |
| **HAS-BLED 0-2**** |  |  |  |  |  |  | 1.41 (1.25-1.59) | 62% |
| **CPRD** |  |  |  |  |  |  |  |  |
| DOACs + interacting antiarrhythmics | 4,679 | 110 | 3,486 | 31.55 | 1.66 (1.12-2.45) | 1.93 (1.29-2.90) |  |  |
| DOACs + non interacting antiarrhythmics | 2,509 | 33 | 1,729 | 19.09 | 1.00 (reference) | 1.00 (reference) |  |  |
| **RAMQ** |  |  |  |  |  |  |  |  |
| DOACs + interacting antiarrhythmics | 16,992 | 1,177 | 26,887 | 43.78 | 1.97 (1.71-2.27) | 1.36 (1.20-1.55) |  |  |
| DOACs + non interacting antiarrhythmics | 6,499 | 227 | 10,265 | 22.11 | 1.00 (reference) | 1.00 (reference) |  |  |
| **HAS-BLED ≥3**** |  |  |  |  |  |  | 1.27 (1.11-1.46) | 69% |
| **CPRD** |  |  |  |  |  |  |  |  |
| DOACs + interacting antiarrhythmics | 7,808 | 298 | 6,445 | 46.24 | 1.30 (0.99-1.72) | 1.04 (0.80-1.35) |  |  |
| DOACs + non interacting antiarrhythmics | 1,824 | 59 | 1,702 | 34.67 | 1.00 (reference) | 1.00 (reference) |  |  |
| **RAMQ** |  |  |  |  |  |  |  |  |
| DOACs + interacting antiarrhythmics | 11,512 | 1,196 | 15,589 | 76.72 | 1.80 (1.52-2.14) | 1.37 (1.17-1.61) |  |  |
| DOACs + non interacting antiarrhythmics | 2,255 | 148 | 3,684 | 40.17 | 1.00 (reference) | 1.00 (reference) |  |  |
| **Apixaban** |  |  |  |  |  |  | 1.32 (1.15-1.51) | 0% |
| **CPRD** |  |  |  |  |  |  |  |  |
| Apixaban + interacting antiarrhythmics | 5,632 | 158 | 4,098 | 38.55 | 1.59 (1.08-2.34) | 1.22 (0.86-1.73) |  |  |
| Apixaban + non interacting antiarrhythmics | 1,738 | 31 | 1,292 | 23.99 | 1.00 (reference) | 1.00 (reference) |  |  |
| **RAMQ** |  |  |  |  |  |  |  |  |
| Apixaban + interacting antiarrhythmics | 14,903 | 1,096 | 19,082 | 57.44 | 2.01 (1.70-2.39) | 1.34 (1.15-1.55) |  |  |
| Apixaban + non interacting antiarrhythmics | 3,876 | 152 | 5,547 | 27.40 | 1.00 (reference) | 1.00 (reference) |  |  |
| **Rivaroxaban** |  |  |  |  |  |  | 1.31 (1.15-1.50) | 83% |
| **CPRD** |  |  |  |  |  |  |  |  |
| Rivaroxaban + interacting antiarrhythmics | 5,304 | 192 | 4,383 | 43.80 | 1.43 (1.04-1.96) | 0.98 (0.74-1.29) |  |  |
| Rivaroxaban + non interacting antiarrhythmics | 1,983 | 38 | 1,559 | 30.79 | 1.00 (reference) | 1.00 (reference) |  |  |
| **RAMQ** |  |  |  |  |  |  |  |  |
| Rivaroxaban + interacting antiarrhythmics | 9,564 | 858 | 14,072 | 60.97 | 2.23 (1.87-2.67) | 1.44 (1.24-1.68) |  |  |
| Rivaroxaban + non interacting antiarrhythmics | 3,395 | 143 | 5,346 | 26.75 | 1.00 (reference) | 1.00 (reference) |  |  |
| **New DOAC use** |  |  |  |  |  |  | 1.50 (1.32-1.72) | 0% |
| **CPRD** |  |  |  |  |  |  |  |  |
| DOACs + interacting antiarrhythmics | 4,006 | 161 | 3,258 | 49.41 | 2.22 (1.44-3.44) | 1.57 (1.08-2.29) |  |  |
| DOACs + non interacting antiarrhythmics | 1,238 | 23 | 1,046 | 21.99 | 1.00 (reference) | 1.00 (reference) |  |  |
| **RAMQ** |  |  |  |  |  |  |  |  |
| DOACs + interacting antiarrhythmics | 15,550 | 1,348 | 22,633 | 59,56 | 2,18 (1.86-2.56) | 1.49 (1.30-1.72) |  |  |
| DOACs + non interacting antiarrhythmics | 4,049 | 172 | 6,511 | 26,42 | 1.00 (reference) | 1.00 (reference) |  |  |
| **Prevalent DOAC use** |  |  |  |  |  |  | 1.02 (0.86-1.21) | 0% |
| **CPRD** |  |  |  |  |  |  |  |  |
| DOACs + interacting antiarrhythmics | 8,481 | 247 | 6,673 | 37.01 | 1.28 (0.98-1.67) | 0.95 (0.75-1.21) |  |  |
| DOACs + non interacting antiarrhythmics | 3,095 | 69 | 2,384 | 28.94 | 1.00 (reference) | 1.00 (reference) |  |  |
| **RAMQ** |  |  |  |  |  |  |  |  |
| DOACs + interacting antiarrhythmics | 12,954 | 1,025 | 19,844 | 51.65 | 1.87 (1.61-2.17) | 1.19 (1.04-1.35) |  |  |
| DOACs + non interacting antiarrhythmics | 4,705 | 203 | 7,438 | 27.29 | 1.00 (reference) | 1.00 (reference) |  |  |

^*^ IR per 1,000 PY. ^**^ HAS-BLED did not include labile international normalized ratio due to lack of standardized reporting in the CPRD and lack of laboratory values in the RAMQ.

Abbreviations: DOACs, direct oral anticoagulants; NVAF, non-valvular atrial fibrillation; PY, patient years; IR, incidence rate; HR, hazard ratio; CI, confidence interval; IPTW, inverse probability of treatment weighting; CPRD, Clinical Practice Research Datalink; RAMQ, Régie de l'Assurance-Maladie du Québec; HAS-BLED, hypertension, abnormal renal or liver function, stroke, bleeding, elderly, drugs or excess alcohol use.

**Table S9. Risk of ischemic stroke associated with concomitant use of DOACs and interacting antiarrhythmics compared with concomitant use of DOACs and non-interacting antiarrhythmics (sensitivity analyses)**

|  | **N**  **Patients** | **N**  **Events** | **N**  **PY** | **IR^*^** | **Crude HR**  **(95%CI)** | **IPTW HR**  **(95%CI)** | **Pooled HR**  **(95% CI)** | **I^2^** |
| --- | --- | --- | --- | --- | --- | --- | --- | --- |
| **15-day grace period** |  |  |  |  |  |  | 0.95 (0.80-1.13) | 0% |
| **CPRD** |  |  |  |  |  |  |  |  |
| DOACs + interacting antiarrhythmics | 12,487 | 67 | 6,625 | 10.11 | 2.06 (1.09-3.89) | 0.97 (0.60-1.56) |  |  |
| DOACs + non interacting antiarrhythmics | 4,333 | 11 | 2,240 | 4.91 | 1.00 (reference) | 1.00 (reference) |  |  |
| **RAMQ** |  |  |  |  |  |  |  |  |
| DOACs + interacting antiarrhythmics | 28,504 | 495 | 33,993 | 14.56 | 1.42 (1.15-1.75) | 0.94 (0.78-1.14) |  |  |
| DOACs + non interacting antiarrhythmics | 8,754 | 105 | 10,204 | 10.29 | 1.00 (reference) | 1.00 (reference) |  |  |
| **Stricter outcome definition** |  |  |  |  |  |  | 1.01 (0.83-1.24) | 0% |
| **CPRD** |  |  |  |  |  |  |  |  |
| DOACs + interacting antiarrhythmics | 12,487 | 65 | 10,131 | 6.42 | 1.59 (0.90-2.84) | 0.90 (0.57-1.43) |  |  |
| DOACs + non interacting antiarrhythmics | 4,333 | 14 | 3,484 | 4.02 | 1.00 (reference) | 1.00 (reference) |  |  |
| **RAMQ** |  |  |  |  |  |  |  |  |
| DOACs + interacting antiarrhythmics | 28,504 | 351 | 44,117 | 7.96 | 1.47 (1.15-1.89) | 1.04 (0.83-1.31) |  |  |
| DOACs + non interacting antiarrhythmics | 8,754 | 76 | 14,218 | 5.35 | 1.00 (reference) | 1.00 (reference) |  |  |
| **Inclusion of fatal events** |  |  |  |  |  |  | 1.06 (0.88-1.27) | 0% |
| **CPRD** |  |  |  |  |  |  |  |  |
| DOACs + interacting antiarrhythmics | 12,487 | 95 | 10,119 | 9,39 | 2.03 (1.20-3.45) | 1.08 (0.71-1.63) |  |  |
| DOACs + non interacting antiarrhythmics | 4,333 | 16 | 3,484 | 4.59 | 1.00 (reference) | 1.00 (reference) |  |  |
| **RAMQ^**^** |  |  |  |  |  |  |  |  |
| DOACs + interacting antiarrhythmics | 21,346 | 426 | 28,387 | 15.01 | 1.61 (1.27-2.02) | 1.05 (0.86-1.30) |  |  |
| DOACs + non interacting antiarrhythmics | 6,721 | 86 | 9,293 | 9.25 | 1.00 (reference) | 1.00 (reference) |  |  |
| **Exclusion of prior events** |  |  |  |  |  |  | 1.03 (0.86-1.23) | 0% |
| **CPRD** |  |  |  |  |  |  |  |  |
| DOACs + interacting antiarrhythmics | 10,633 | 55 | 8,491 | 6.48 | 2.19 (1.08-4.42) | 0.92 (0.56-1.52) |  |  |
| DOACs + non interacting antiarrhythmics | 3,853 | 9 | 3,043 | 2.96 | 1.00 (reference) | 1.00 (reference) |  |  |
| **RAMQ** |  |  |  |  |  |  |  |  |
| DOACs + interacting antiarrhythmics | 25,965 | 481 | 40,331 | 11.93 | 1.74 (1.39-2.17) | 1.05 (0.86-1.27) |  |  |
| DOACs + non interacting antiarrhythmics | 8,142 | 90 | 13,267 | 6.78 | 1.00 (reference) | 1.00 (reference) |  |  |
| **Multiple imputation^***^** |  |  |  |  |  |  |  |  |
| **CPRD** |  |  |  |  |  |  |  |  |
| DOACs + interacting antiarrhythmics | 12,478 | 92 | 10,119 | 9,09 | 1.97 (1.16-3.35) | 1.02 (0.67-1.54) |  |  |
| DOACs + non interacting antiarrhythmics | 4,333 | 16 | 3,484 | 4,59 | 1.00 (reference) | 1.00 (reference) |  |  |
| **Intention-to-treat** |  |  |  |  |  |  | 1.01 (0.85-1.21) | 71% |
| **CPRD** |  |  |  |  |  |  |  |  |
| DOACs + interacting antiarrhythmics | 12,487 | 123 | 10,560 | 11.65 | 2.19 (1.37-3.52) | 1.44 (0.96-2.17) |  |  |
| DOACs + non interacting antiarrhythmics | 4,333 | 20 | 3,776 | 5.30 | 1.00 (reference) | 1.00 (reference) |  |  |
| **RAMQ** |  |  |  |  |  |  |  |  |
| DOACs + interacting antiarrhythmics | 28,504 | 440 | 24,960 | 17.63 | 1.44 (1.16-1.79) | 0.94 (0.77-1.14) |  |  |
| DOACs + non interacting antiarrhythmics | 8,754 | 98 | 8,087 | 12.12 | 1.00 (reference) | 1.00 (reference) |  |  |
| **IPCW**^****^ |  |  |  |  |  |  | 1.13 (0.93-1.38) | 0% |
| **CPRD** |  |  |  |  |  |  |  |  |
| DOACs + interacting antiarrhythmics | 12,487 | 92 | 10,119 | 9.09 | 1.97 (1.16-3.35) | 1.22 (0.70-2.14) |  |  |
| DOACs + non interacting antiarrhythmics | 4,333 | 16 | 3,484 | 4.59 | 1.00 (reference) | 1.00 (reference) |  |  |
| **RAMQ** |  |  |  |  |  |  |  |  |
| DOACs + interacting antiarrhythmics | 28,504 | 616 | 43,983 | 14.01 | 1.59 (1.31-1.93) | 1.12 (0.91-1.38) |  |  |
| DOACs + non interacting antiarrhythmics | 8,754 | 123 | 14,172 | 8.68 | 1.00 (reference) | 1.00 (reference) |  |  |

^*^ IR per 1,000 PY. ^**^ The study period for this analysis ended on December 31, 2018 due to lack of availability of cause of death in the RAMQ data beyond this date. 9,191 subjects were removed because their cohort entry was after the end date of the study. ^***^ This analysis was conducted only in the CPRD for missing values for body mass index and blood pressure. The HR was pooled from 10 imputed datasets. ^****^ Time-varying covariates included alcohol-related disorders and use of antiplatelet agents. Extreme weights were truncated using the 99th percentile as cut-off.

Abbreviations: DOACs, direct oral anticoagulants; NVAF, non-valvular atrial fibrillation; PY, patient years; IR, incidence rate; HR, hazard ratio; CI, confidence interval; NA, not applicable; IPCW, inverse probability of censoring weighting; CPRD, Clinical Practice Research Datalink; RAMQ, Régie de l'Assurance-Maladie du Québec.

# **Table S10. Risk of major bleeding associated with concomitant use of DOACs and interacting antiarrhythmics compared with concomitant use of DOACs and non-interacting antiarrhythmics (sensitivity analyses)**

|  | **N**  **Patients** | **N**  **Events** | **N**  **PY** | **IR^*^** | **Crude HR**  **(95%CI)** | **IPTW HR**  **(95%CI)** | **Pooled HR**  **(95% CI)** | **I^2^** |
| --- | --- | --- | --- | --- | --- | --- | --- | --- |
| **15-day grace period** |  |  |  |  |  |  | 1.31 (1.19-1.45) | 0% |
| **CPRD** |  |  |  |  |  |  |  |  |
| DOACs + interacting antiarrhythmics | 12,487 | 288 | 6,535 | 44.07 | 1.63 (1.24-2.16) | 1.25 (0.97-1.61) |  |  |
| DOACs + non interacting antiarrhythmics | 4,333 | 60 | 2,220 | 27.02 | 1.00 (reference) | 1.00 (reference) |  |  |
| **RAMQ** |  |  |  |  |  |  |  |  |
| DOACs + interacting antiarrhythmics | 28,504 | 2,001 | 33,023 | 60.59 | 1.98 (1.76-2.23) | 1.33 (1.19-1.47) |  |  |
| DOACs + non interacting antiarrhythmics | 8,754 | 308 | 10,077 | 30.57 | 1.00 (reference) | 1.00 (reference) |  |  |
| **Stricter outcome definition** |  |  |  |  |  |  | 1.08 (0.94-1.24) | 0% |
| **CPRD** |  |  |  |  |  |  |  |  |
| DOACs + interacting antiarrhythmics | 12,487 | 230 | 9,998 | 23.00 | 1.62 (1.19-2.20) | 1.09 (0.84-1.42) |  |  |
| DOACs + non interacting antiarrhythmics | 4,333 | 49 | 3,455 | 14.18 | 1.00 (reference) | 1.00 (reference) |  |  |
| **RAMQ** |  |  |  |  |  |  |  |  |
| DOACs + interacting antiarrhythmics | 28,504 | 677 | 43,768 | 15.47 | 1.50 (1.25-1.80) | 1.08 (0.92-1.27) |  |  |
| DOACs + non interacting antiarrhythmics | 8,754 | 143 | 14,139 | 10.11 | 1.00 (reference) | 1.00 (reference) |  |  |
| **Inclusion of fatal events** |  |  |  |  |  |  | 1.29 (1.17-1.43) | 66% |
| **CPRD** |  |  |  |  |  |  |  |  |
| DOACs + interacting antiarrhythmics | 12,487 | 412 | 9,931 | 41.49 | 1.53 (1.22-1.91) | 1.11 (0.91-1.36) |  |  |
| DOACs + non interacting antiarrhythmics | 4,333 | 93 | 3,431 | 27.11 | 1.00 (reference) | 1.00 (reference) |  |  |
| **RAMQ^**^** |  |  |  |  |  |  |  |  |
| DOACs + interacting antiarrhythmics | 21,346 | 1,620 | 27,585 | 58.73 | 2.02 (1.77-2.30) | 1.36 (1.21-1.53) |  |  |
| DOACs + non interacting antiarrhythmics | 6,721 | 262 | 9,174 | 28.56 | 1.00 (reference) | 1.00 (reference) |  |  |
| **Exclusion of prior events** |  |  |  |  |  |  | 1.31 (1.19-1.44) | 0% |
| **CPRD** |  |  |  |  |  |  |  |  |
| DOACs + interacting antiarrhythmics | 12,022 | 361 | 9,605 | 37.59 | 1.62 (1.27-2.07) | 1.28 (1.02-1.60) |  |  |
| DOACs + non interacting antiarrhythmics | 4,231 | 78 | 3,364 | 23.19 | 1.00 (reference) | 1.00 (reference) |  |  |
| **RAMQ** |  |  |  |  |  |  |  |  |
| DOACs + interacting antiarrhythmics | 24,460 | 1,823 | 37,248 | 48.94 | 1.93 (1.71-2.18) | 1.32 (1.18-1.47) |  |  |
| DOACs + non interacting antiarrhythmics | 7,919 | 316 | 12,695 | 24.89 | 1.00 (reference) | 1.00 (reference) |  |  |
| **Multiple imputation^***^** |  |  |  |  |  |  | NA |  |
| **CPRD** |  |  |  |  |  |  |  |  |
| DOACs + interacting antiarrhythmics | 12,487 | 408 | 9,931 | 41.08 | 1.53 (1.22-1.92) | 1.11 (0.91-1.36) |  |  |
| DOACs + non interacting antiarrhythmics | 4,333 | 92 | 3,431 | 26.82 | 1.00 (reference) | 1.00 (reference) |  |  |
| **Intention-to-treat** |  |  |  |  |  |  | 1.31 (1.19-1.45) | 86% |
| **CPRD** |  |  |  |  |  |  |  |  |
| DOACs + interacting antiarrhythmics | 12,487 | 451 | 10,421 | 43.28 | 1.66 (1.33-2.07) | 1.05 (0.87-1.27) |  |  |
| DOACs + non interacting antiarrhythmics | 4,333 | 97 | 3,733 | 25.99 | 1.00 (reference) | 1.00 (reference) |  |  |
| **RAMQ** |  |  |  |  |  |  |  |  |
| DOACs + interacting antiarrhythmics | 28,504 | 1,804 | 24,263 | 74.35 | 2.15 (1.89-2.44) | 1.42 (1.27-1,59) |  |  |
| DOACs + non interacting antiarrhythmics | 8,754 | 273 | 7,998 | 34.14 | 1.00 (reference) | 1.00 (reference) |  |  |
| **IPCW^****^** |  |  |  |  |  |  | 1.42 (1.28-1,57) | 86% |
| **CPRD** |  |  |  |  |  |  |  |  |
| DOACs + interacting antiarrhythmics | 12,487 | 408 | 9,931 | 41.08 | 1.53 (1.22-1.92) | 1.06 (0.83-1.34) |  |  |
| DOACs + non interacting antiarrhythmics | 4,333 | 92 | 3,431 | 26.82 | 1.00 (reference) | 1.00 (reference) |  |  |
| **RAMQ** |  |  |  |  |  |  |  |  |
| DOACs + interacting antiarrhythmics | 28,504 | 2,373 | 42,477 | 55.87 | 2.03 (1.82-2.27) | 1.52 (1.35-1.70) |  |  |
| DOACs + non interacting antiarrhythmics | 8,754 | 375 | 13,949 | 26.88 | 1.00 (reference) | 1.00 (reference) |  |  |

^*^ IR per 1,000 PY. ^**^ The study period for this analysis ended on December 31, 2018 due to lack of availability of cause of death in the RAMQ data beyond this date. 9,191 subjects were removed because their cohort entry was after the end date of the study. ^***^ This analysis was conducted only in the CPRD for missing values for body mass index and blood pressure. The HR was pooled from 10 imputed datasets. ^****^ Time-varying covariates included alcohol-related disorders, use of antiplatelet agents, and use non-steroidal anti-inflammatory drugs. Extreme weights were truncated using the 99th percentile as cut-off.

Abbreviations: DOACs, direct oral anticoagulants; NVAF, non-valvular atrial fibrillation; PY, patient years; IR, incidence rate; HR, hazard ratio; CI, confidence interval; NA, not applicable; MSM, marginal structural model; IPCW, inverse probability of censoring weighting; CPRD, Clinical Practice Research Datalink; RAMQ, Régie de l'Assurance-Maladie du Québec.
